# Supplementary material for: A meta-analysis of the ecological and economic outcomes of mangrove restoration
Source: Nat Commun. 2021 Aug 19;12:5050. doi: 10.1038/s41467-021-25349-1 (PMC8376958; doi:10.1038/s41467-021-25349-1)
Supplement: Supplementary file 1 — Supplementary Information [file 41467_2021_25349_MOESM1_ESM.pdf]

Supplementary Information for

A meta-analysis of the ecological and economic outcomes of mangrove restoration

Jie Su [1*], Daniel A. Friess [2, 3], Alexandros Gasparatos [4,5]

1 - Graduate Program in Sustainability Science - Global Leadership Initiative (GPSS-GLI), Graduate School of Frontier Sciences, The University of Tokyo, 5-1-5 Kashiwanoha, Kashiwa City, 277-8563, Japan
2 - Department of Geography, National University of Singapore, 1 Arts Link, Singapore 117570
3 - Centre for Nature-based Climate Solutions, National University of Singapore, 16 Science Drive 4, Singapore 117558
4 - Institute for Future Initiatives (IFI), The University of Tokyo, 7-3-1 Hongo, Bunkyo-ku, Tokyo, 113-8654, Japan
5 - Institute for the Advanced Study of Sustainability (UNU-IAS), United Nations University, 5-53-70 Jingumae, Shibuya-ku, Tokyo 150-8925, Japan

*Corresponding author. Email address: jie.su@s.k.u-tokyo.ac.jp

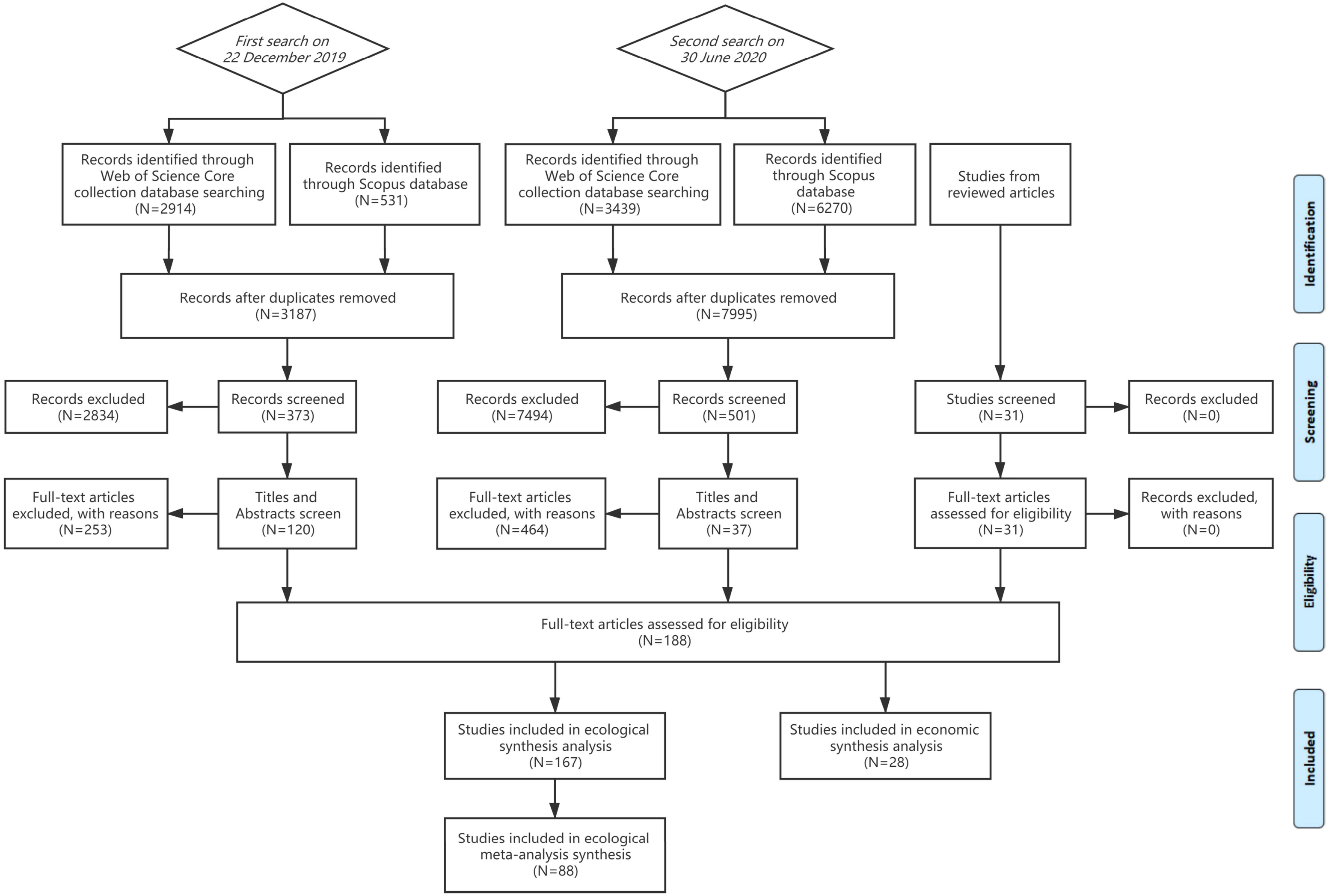

Supplementary Figure 1. PRISMA flow diagram for the studies selected and included in the systematic review.

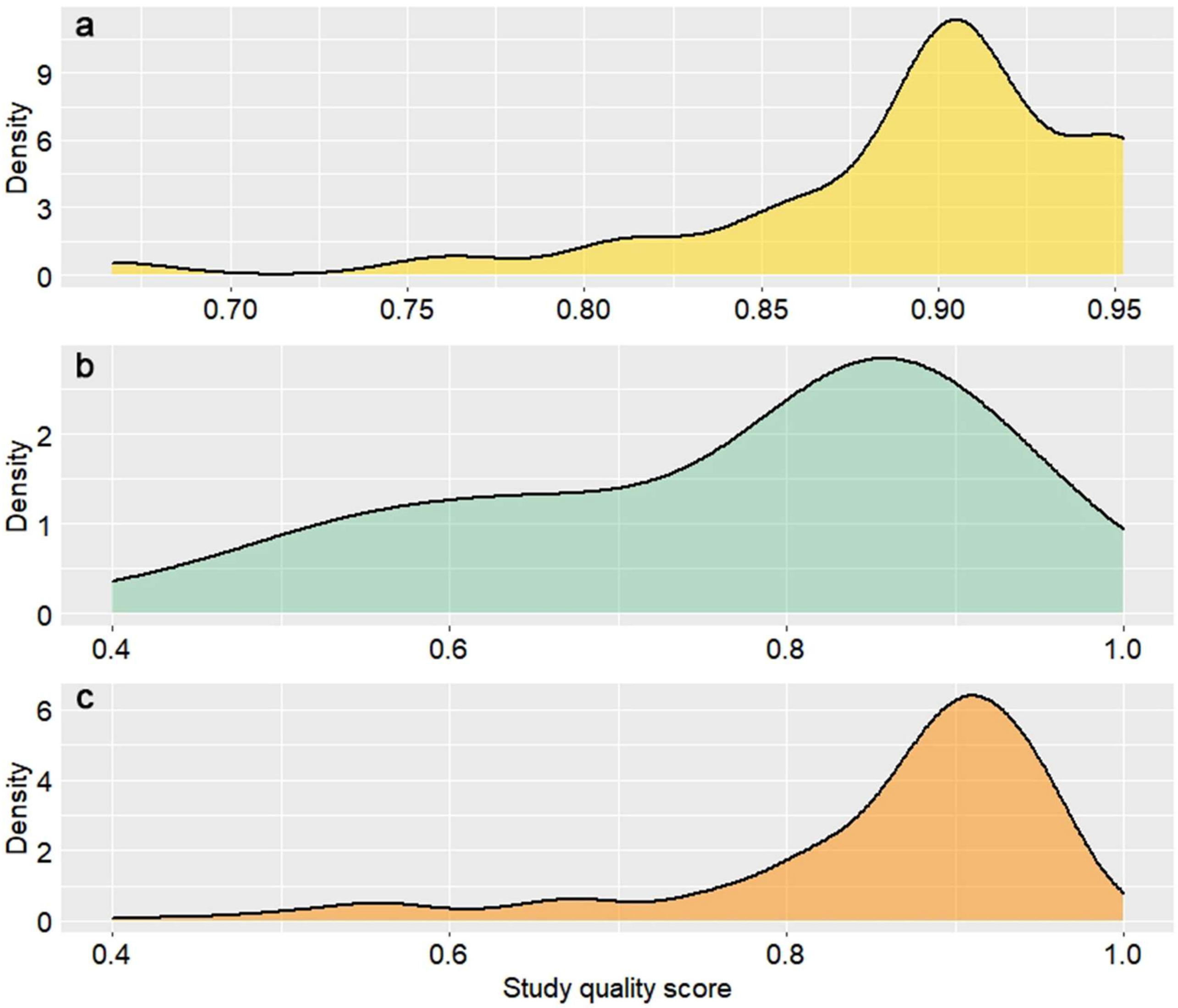

Supplementary Figure 2. Kernel density of study quality scores for studies. Panel **a** the meta-analysis (N=88), **b** economic analysis (N=28), and **c** systematic review (N=188). Source data are provided as a Source Data file.

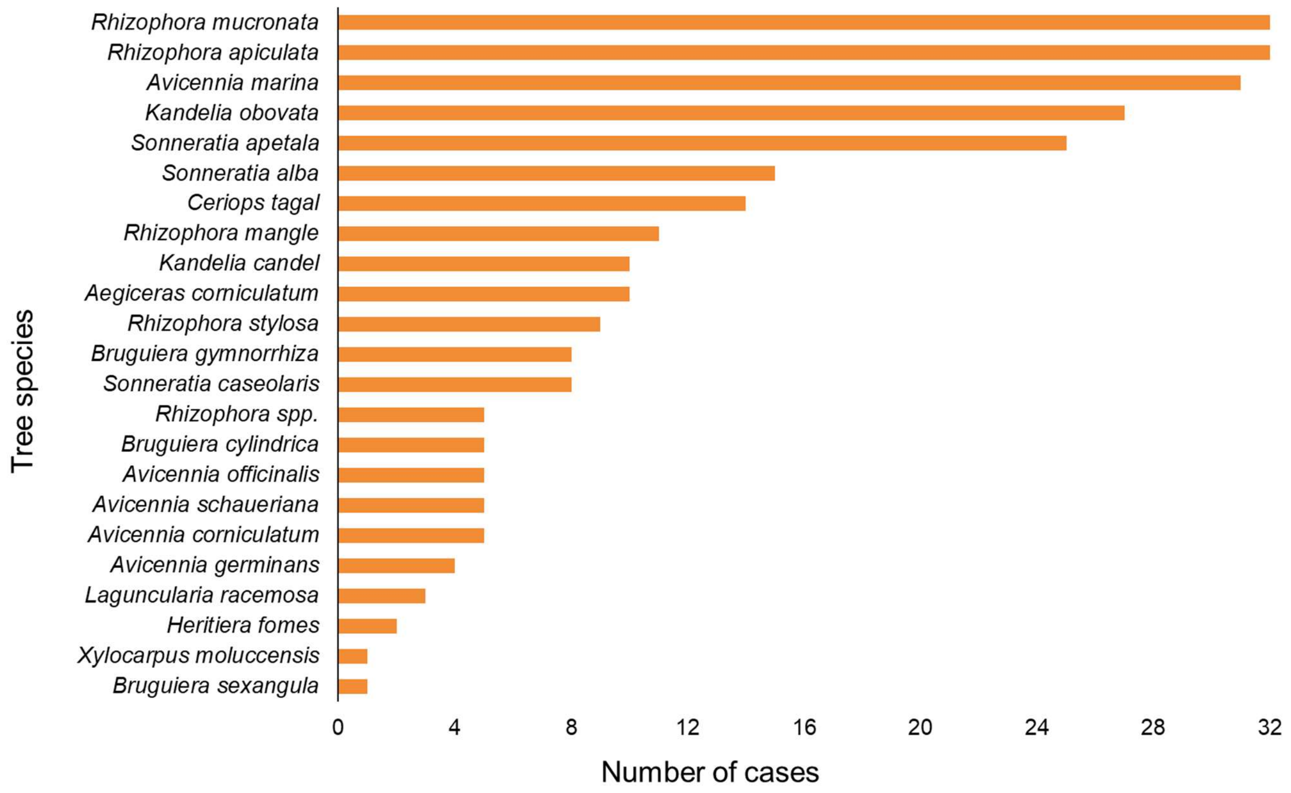

Supplementary Figure 3. Tree species used for mangrove restoration. Source data are provided as a Source Data file.

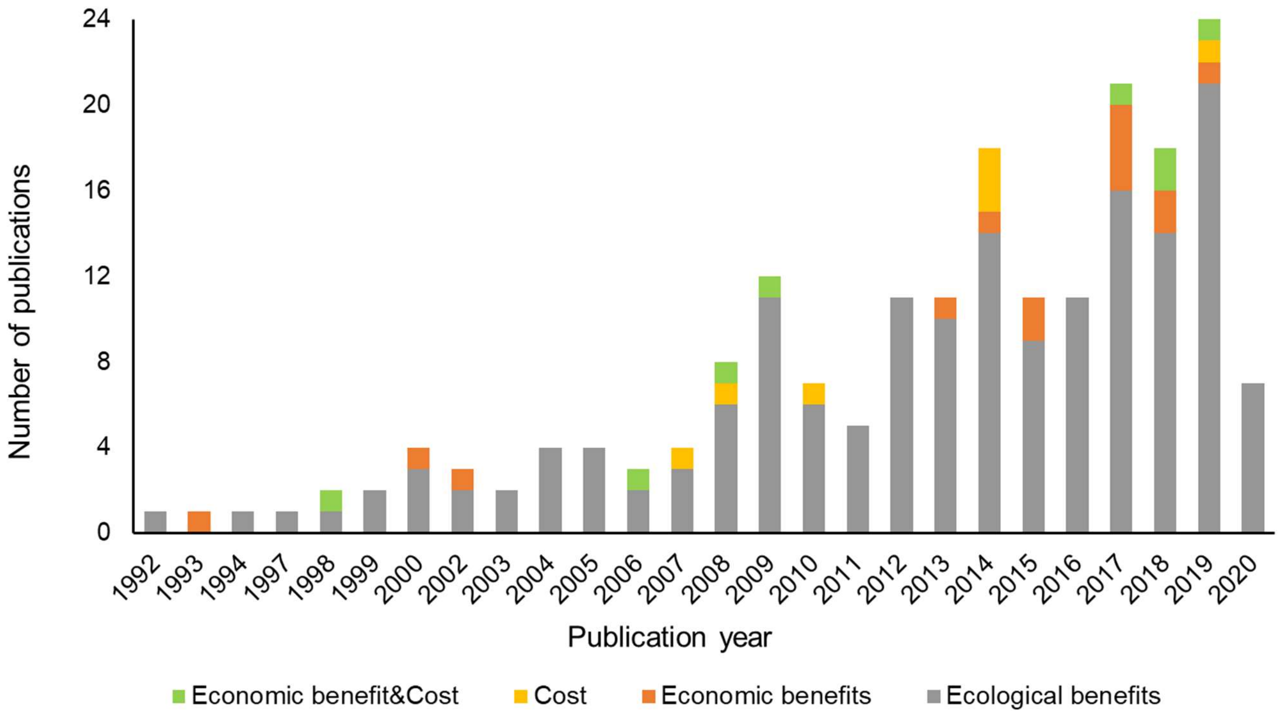

Supplementary Figure 4. Focus of studies across years. Source data are provided as a Source Data file.

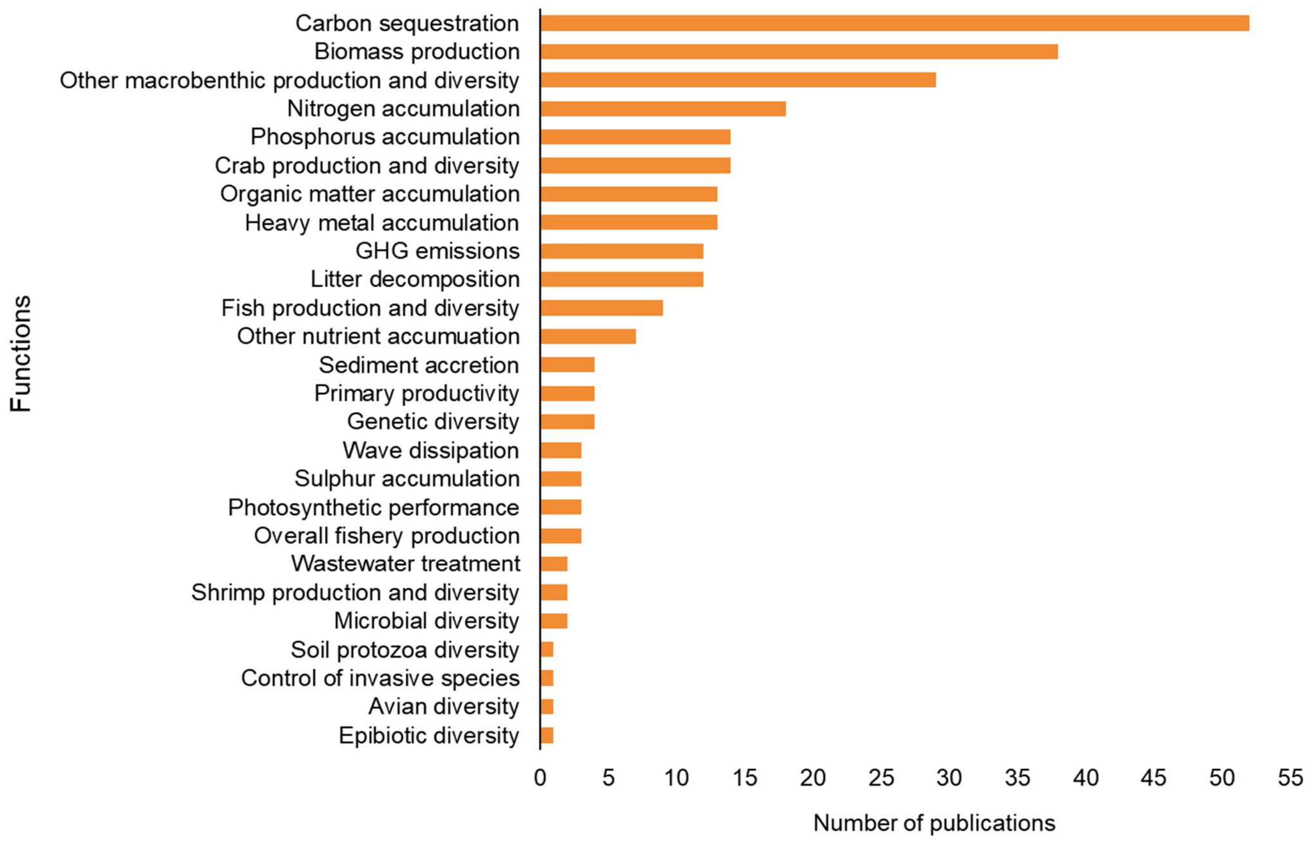

Supplementary Figure 5. Number of publications by considered function. Source data are provided as a Source Data file.

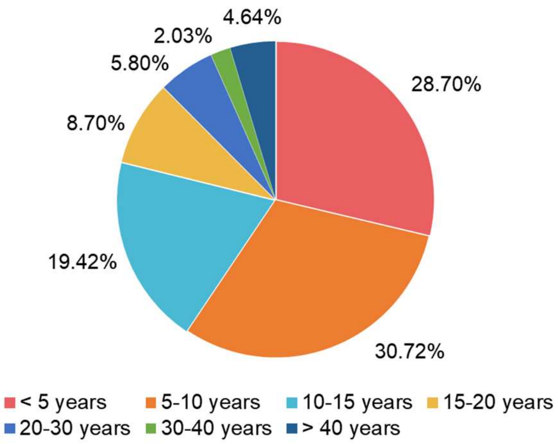

Supplementary Figure 6. Proportion of restoration age groups in total number of cases. Source data are provided as a Source Data file.

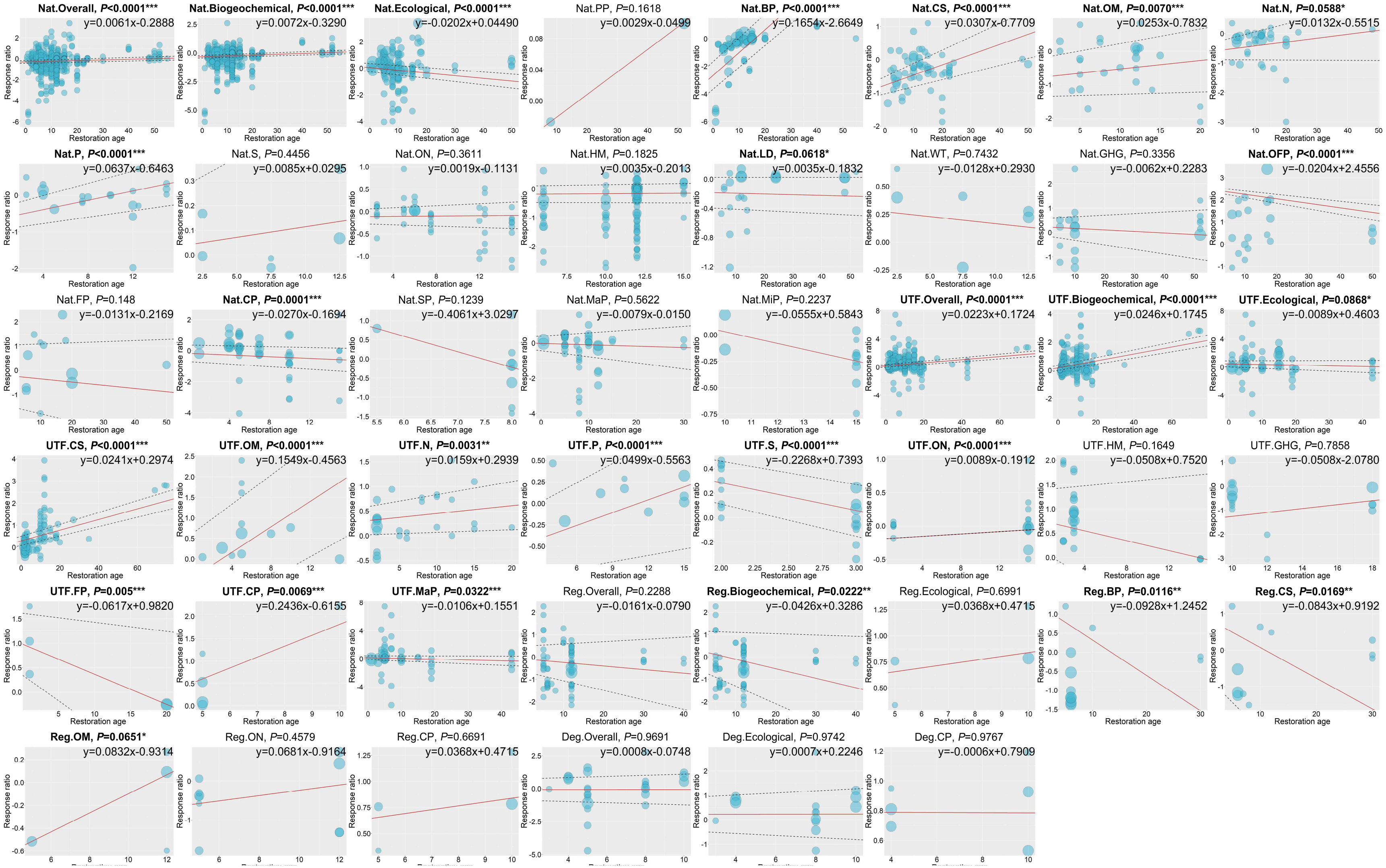

Supplementary Figure 7. Response of restoration outcomes to stand age. Bubble plots illustrate the meta-regression results between the effect size of restoration outcome and the tree age of restored mangroves using one-sided test. The value of *P* is the corresponding p-values of the linear regression model. Dot size is in proportion to weight. Red lines and dashed lines indicate the fits and the 95% confidence intervals for the mixed effects meta regressions. Partial or missing shaded lines suggest large 95%CIs that cannot be shown in the figures. Text in bold indicates statistical significance. Refer to Supplementary Table 1 for function abbreviations; '***' significant at 1% level; '**' significant at 5% level; '*' significant at 10% level. Source data are provided as a Source Data file.

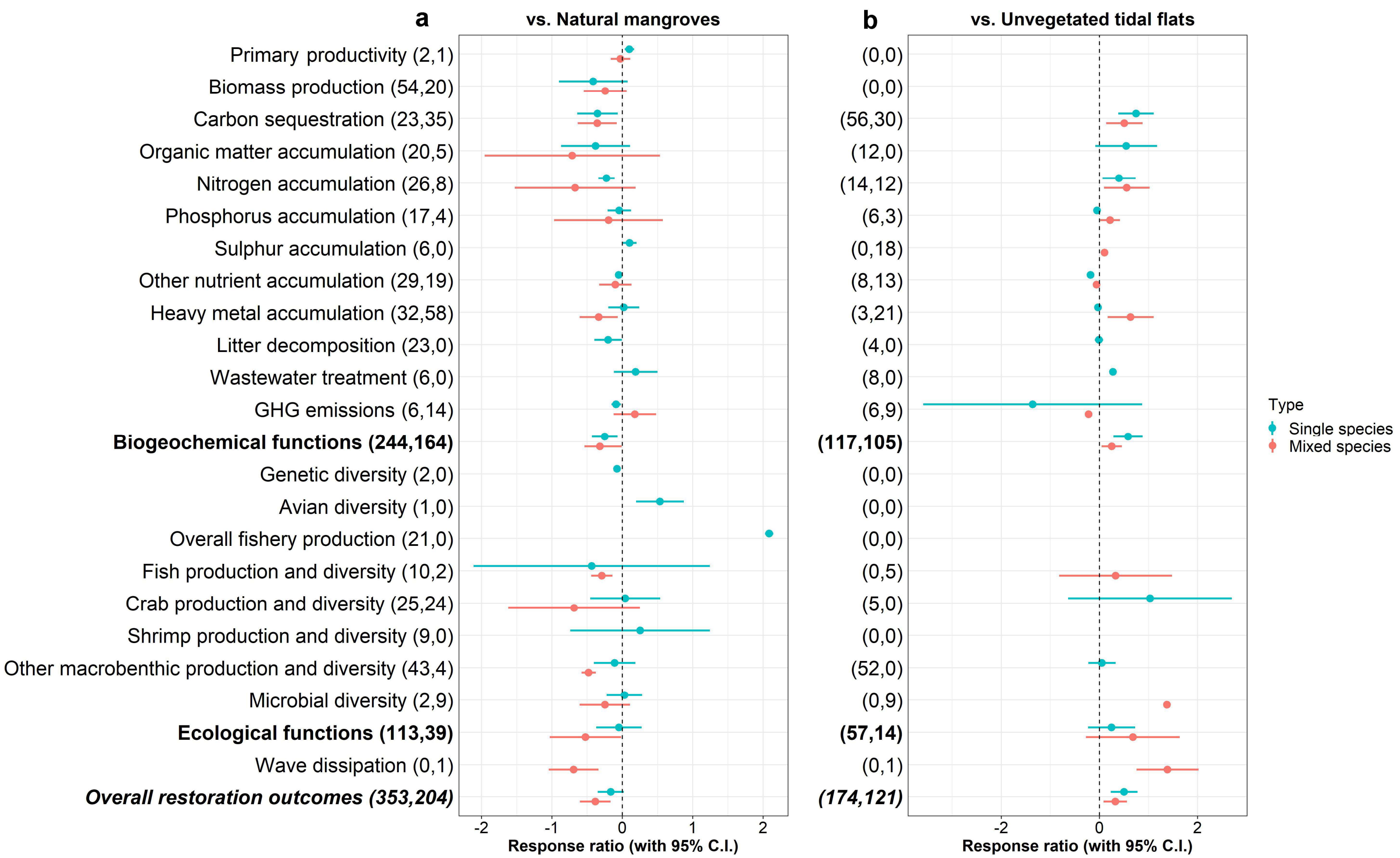

Supplementary Figure 8. Mean effect size for different types of functions with single species and mixed species plantation. Panel **a** restored mangroves vs. natural mangroves, **b** restored mangroves vs. unvegetated tidal flats. Bars around the means denote 95% confidence intervals. If a bar falls in the positive side and does not intersect with zero we interpret that the restored mangrove provides this specific function at a higher level than the comparative basis, and the opposite if it falls in the negative side of the forest plot. The first and second numbers in parentheses indicate, respectively, the sample size for using single species and the sample size for using mixed species. Source data are provided as a Source Data file.

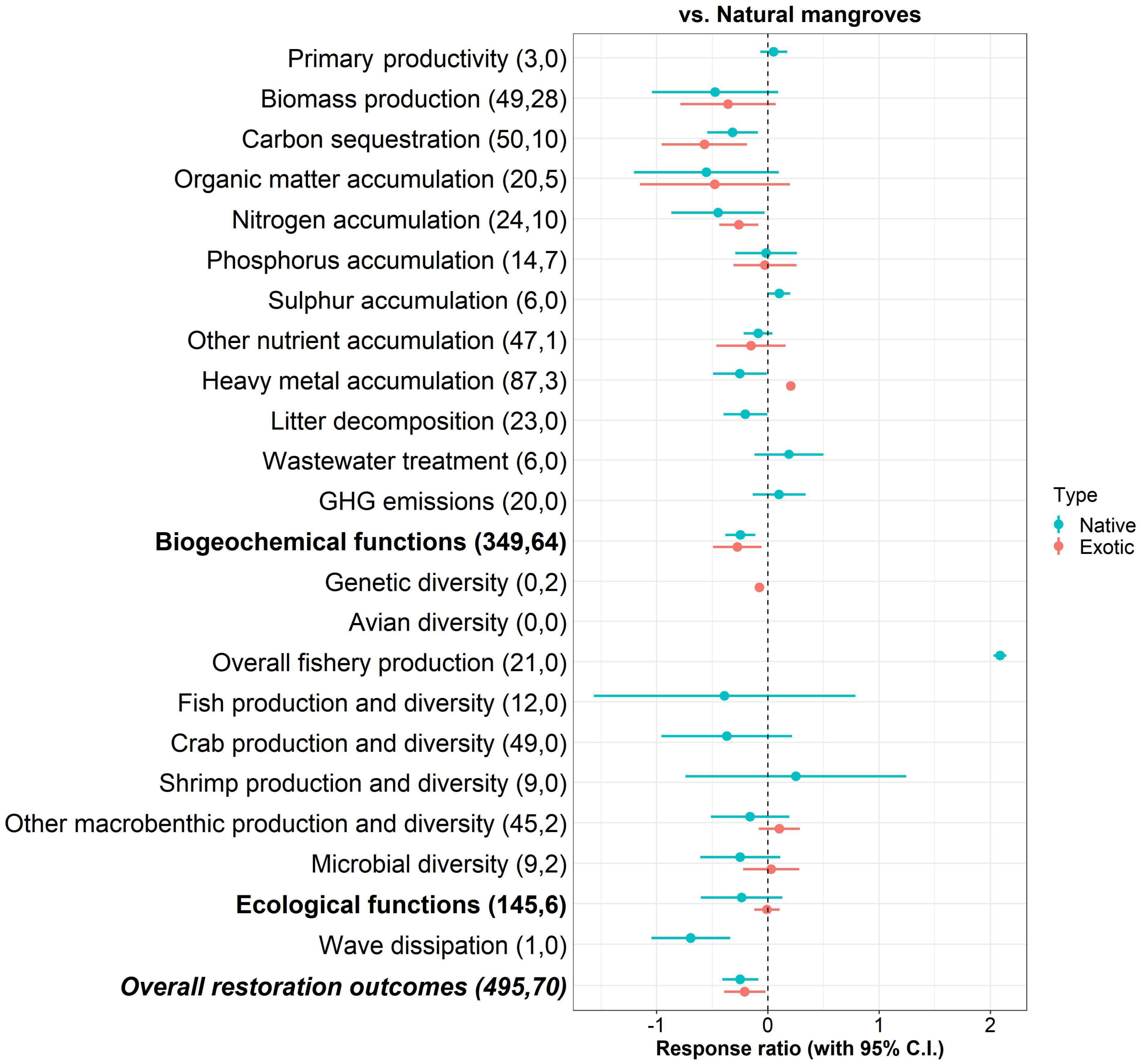

Supplementary Figure 9. Mean effect size for different types of functions with native species and exotic species plantation for restored mangroves vs. natural mangroves. Bars around the means denote 95% confidence intervals. If a bar falls in the positive side and does not intersect with zero we interpret that the restored mangrove provides this specific function at a higher level than the comparative basis, and the opposite if it falls in the negative side of the forest plot. The first and second numbers in parentheses indicate, respectively, the sample size for using native species and the sample size for using exotic species. Source data are provided as a Source Data file.

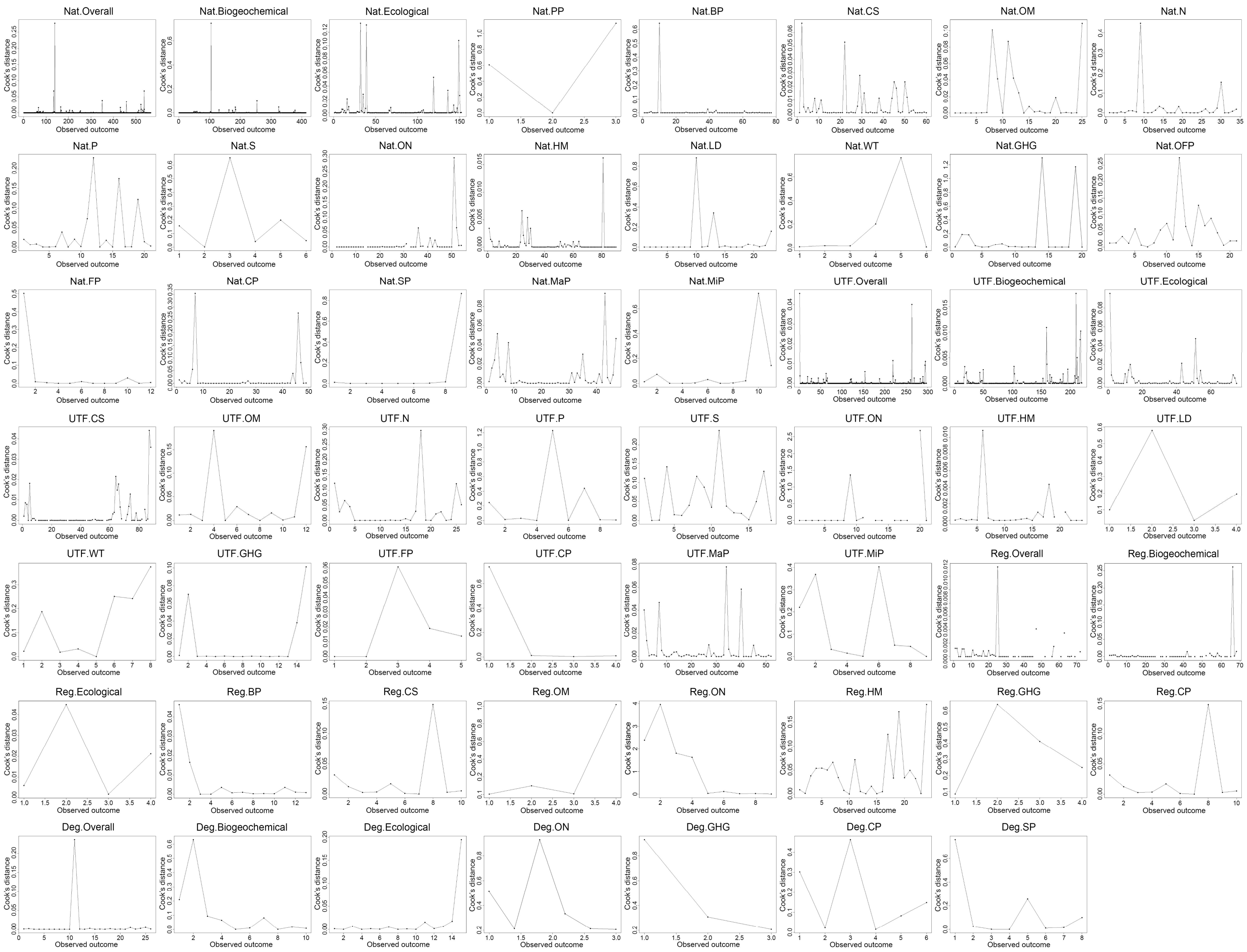

Supplementary Figure 10. Cook's distance diagnoses. Plots detect outliers when a Cook's D is more than traditional threshold of 4/*n* (*n*=sample size)[1]. Refer to Supplementary Table 1 for function abbreviations. Source data are provided as a Source Data file.

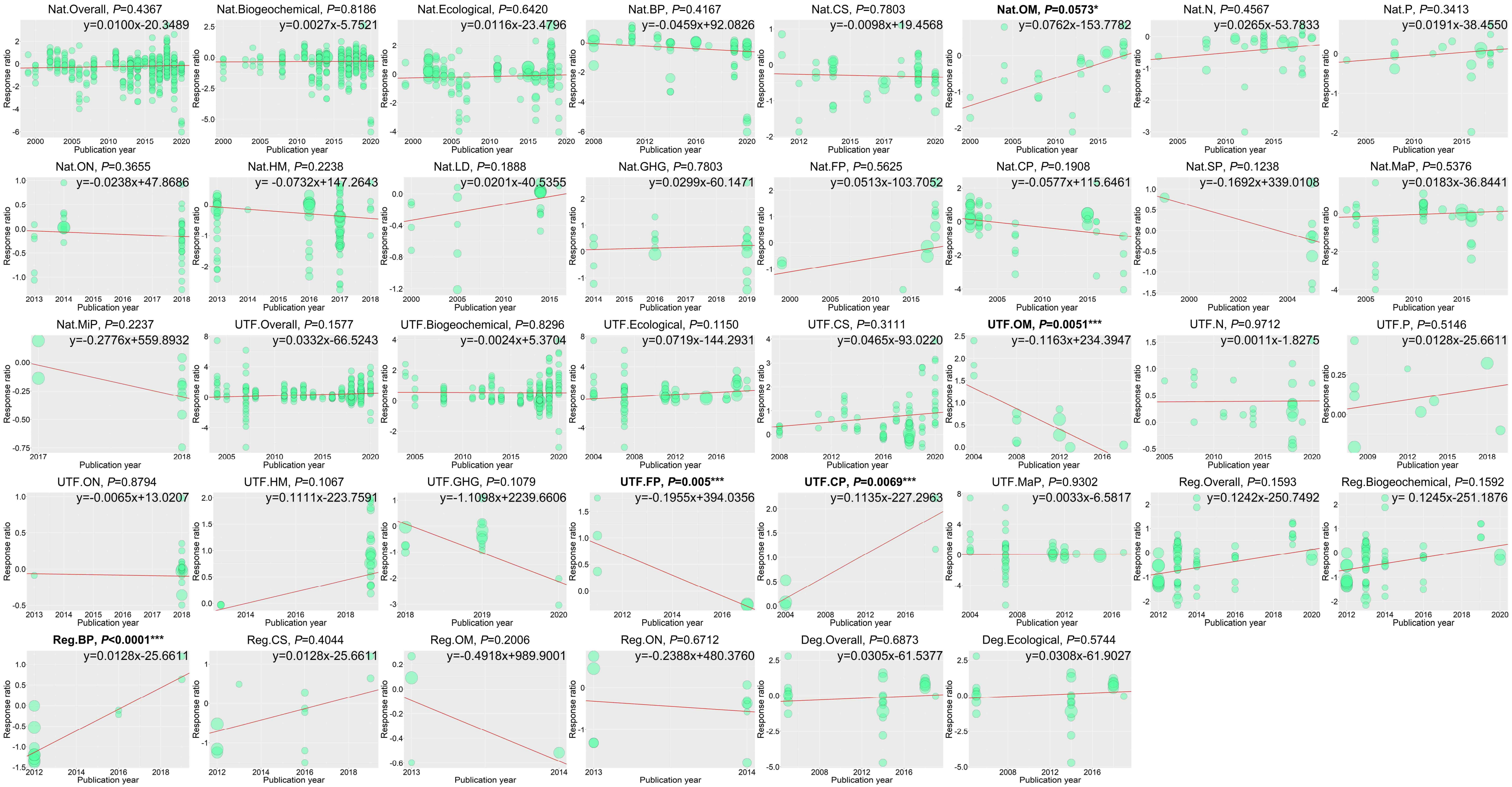

Supplementary Figure 11. Temporal change test. Bubble plots illustrate the meta-regression results between the effect size of aggregated outcomes and publication year using one-sided test. The value of *P* is the corresponding p-values of the linear regression model. Dot sizes are in proportion to weight. Red lines indicate the fits for the mixed effects meta regressions. Refer to Supplementary Table 1 for function abbreviations. Text in bold indicates statistical significance: '***' significant at 1% level; '**' significant at 5% level; '*' significant at 10% level. Source data are provided as a Source Data file.

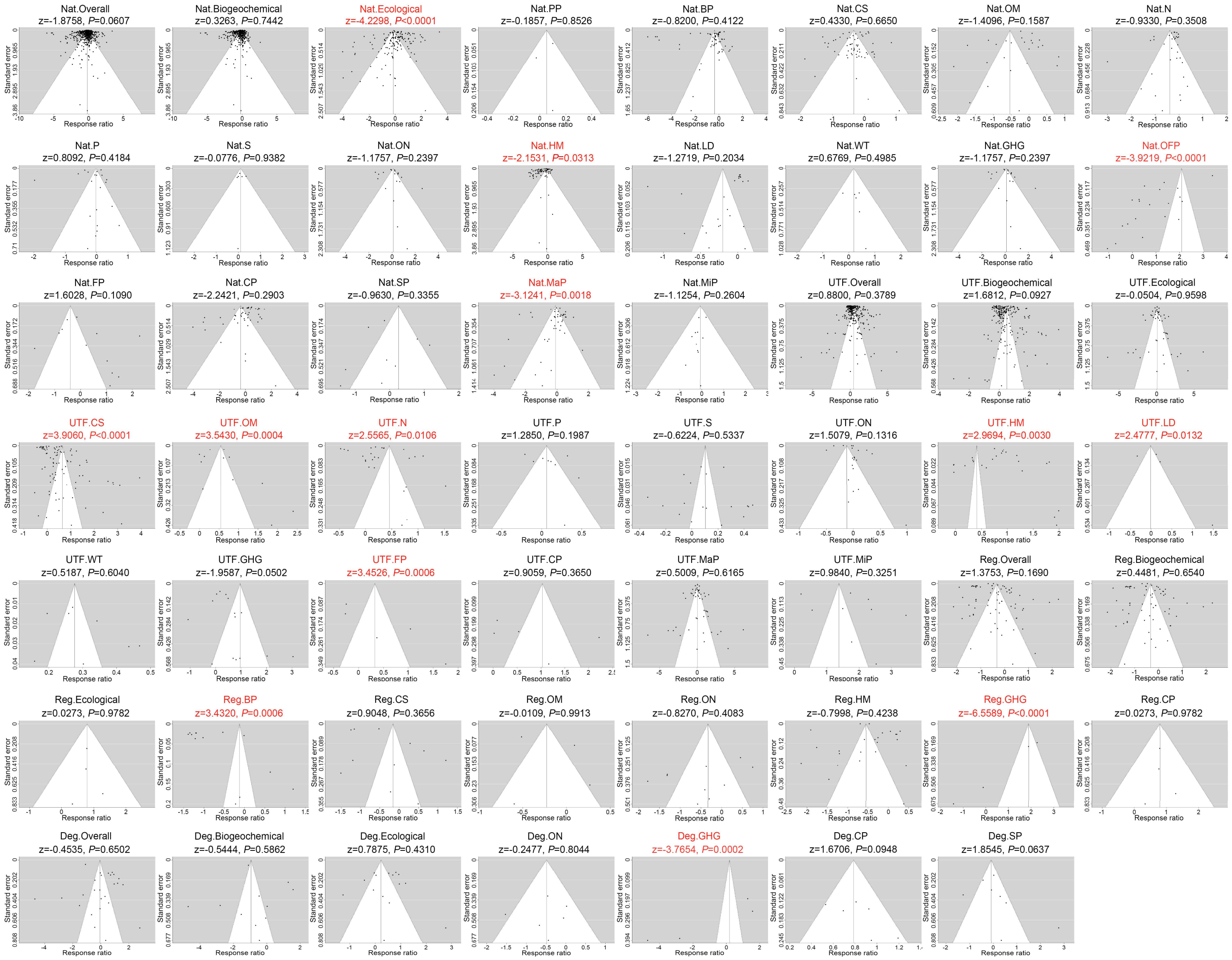

Supplementary Figure 12. Funnel plots of overall restoration outcomes and individual functions. The top of each panel contains the results of publication bias tests using Egger's regression (z and P-value). P<0.05 indicate the presence of publication bias (indicated with text in red). The outer dashed lines indicate the triangular region within which 95% of studies are expected to lie in the absence of both bias and heterogeneity. Refer to Supplementary Table 1 for function abbreviations.

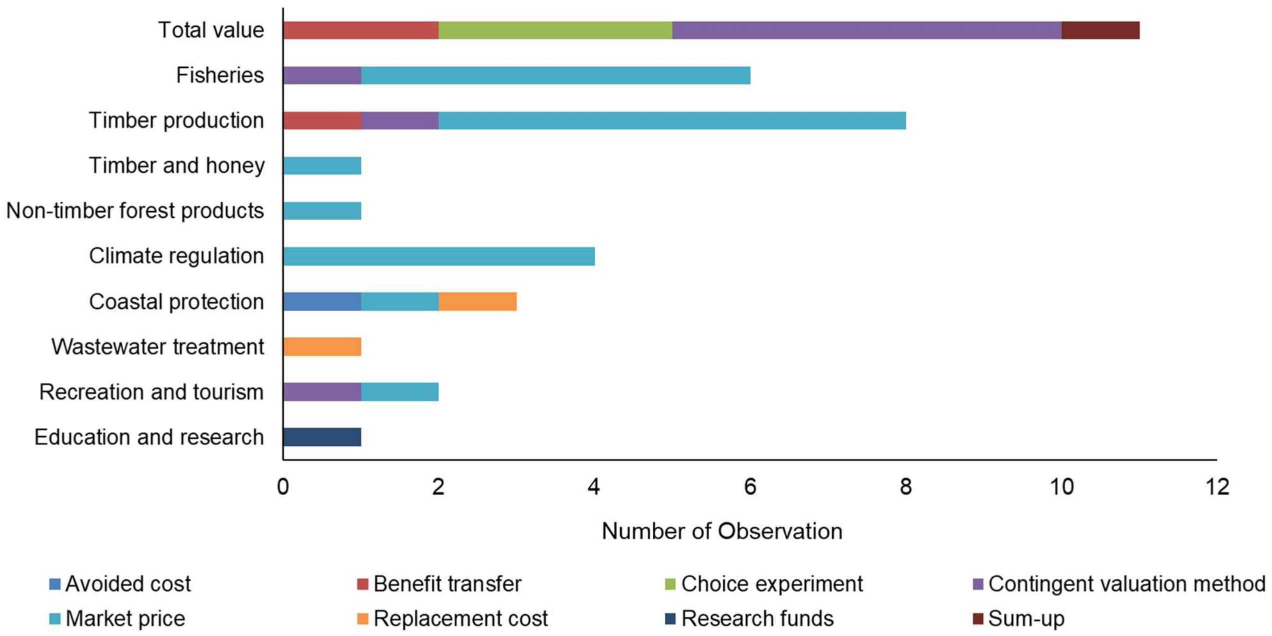

Supplementary Figure 13. Valuation methods for the economic benefits of mangrove restoration. Source data are provided as a Source Data file.

Supplementary Table 1. Ecosystem functions and response variables covered in the systematic review.

Function category	Definition of category[2]	Individual functions	Abbreviation in this study	Variables	Description of function	Estimation method
Biogeochemical functions	Biogeochemical functions include primary and secondary production, decomposition, nutrient cycling, hydrology, soil development and soil fertility, regulation of climate cycles, stabilization of substrates, and purification of the air and water.	Primary productivity	PP	Gross primary productivity, net primary productivity	Mangroves are very productive ecosystems, with the total net primary production including above-ground (i.e. wood. leaves) belowground (i.e. roots) production[3].	Sample collections, measurement and calculation
		Photosynthetic performance	Ph	ERR, Fv/Fm, rETRmax, OptE, bETR, aETR, Chlorophyll	Photosynthetic performance/efficiency refers to the amount of light energy plants and algae that are able to convert into chemical energy through photosynthesis.	Sampling and measurement
		Biomass production	BP	Aboveground biomass, belowground biomass, total biomass, carbon in biomass	Mangroves have high above-ground biomass and overall carbon storage capacity, due to their large, woody growth forms[4].	Plot design and measurement
		Carbon sequestration	CS	Soil organic carbon, total carbon stock		Plot design and measurement
		Organic matter accumulation	OM	Organic matter content	Mangroves generate significant quantities of organic matter (OM) via litterfall that can be transported offshore[5].	Sample collection and measurement
		Nitrogen accumulation	N	N concentration, N fixation, NH4-N, C:N ratio	Nitrogen, phosphorus, and sulphur are three major macronutrients that are essential for various biological activities. Mangroves are regarded as sinks of sediments and nutrients that could accumulate nutrients[6]. Here we classify nutrient accumulation into nitrogen accumulation, phosphorus accumulation, sulphur accumulation and other nutrient accumulation.	Sample collection and measurement
		Phosphorus accumulation	P	P concentration, PO4-P		Sample collection and measurement
		Sulphur accumulation	S	S concentration, H2O-S, absorbed-S, AS, arylsulfatase contents		Sample collection and measurement
		Other nutrient accumulation	ON	K, Na, Ca, Mg, Al,		Sample collection and measurement
		Heavy metal accumulation	HM	Cu, Mg, Zn, Pb, Cr, Cd, Hg, Ni, Cr, As	Mangrove sediments have high capacity to bind metals due to their anaerobic conditions with the rich sulphide content. This can increase metal concentration in the mangrove sediments, therefore leading to bioaccumulation in plant tissues[7].	Sample collection, retreatment and measurement
		Litter decomposition	LD	Productivity rates, annual litter fall, C return, N return, P return	Litter decomposition is a process in which organic matter produced by mangrove forests is transferred to the sediment, and it consists at least of four basic steps: (a) removal of soluble compounds of leaves by water, (b) colonization by microorganisms, (c) consumption of vegetal matter by herbivores, and (d) mixed action of physical forces[8].	Sample collection and measurement
		Wastewater treatment	WT	COD, BOD_5, TN, NH3-N, TP, SP, PCBs	Mangrove wetlands can remove nutrients, heavy metals, and organic pollutants from wastewater within estuarine systems due to the presence of oxidized and reduced conditions, periodic flooding by incoming and outgoing tides, and high clay and organic matter content[9].	Sample collection and measurement

		GHG emissions	GHG	CO_2, CH_4, N_2O, soil respiration, ecosystem cooling effect, rates of total carbon oxidation	The saline conditions in healthy mangroves emit negligible amounts of other greenhouse gases (GHGs) such as methane (CH_4) and nitrous oxide (N_2O), which are substantially more potent GHGs than CO_2[10]. Considering this is a negative function, we inverted the sign of the RR for those "negative" variables before combining them with other functions to calculate the overall effect size.	Measurement (e.g. photoacoustic infrared gas analyser)
Ecological functions	Ecological functions are those that regulate population dynamics and species composition, such as resilience and resistance to biotic and abiotic perturbations, maintenance of food-web integrity, and provision of habitat for a range of trophic levels.	Genetic diversity	GB	Genetic biodiversity, genetic diversity index (HS), number of alleles	Genetic diversity is the total number of genetic characteristics in the genetic makeup of a species, it ranges widely from the number of species to differences within species and can be attributed to the span of survival for a species.	Sampling and DNA extraction
		Avian diversity	AD	Richness	Mangrove ecosystems are important habitats for diverse bird species[11].	Bird surveys
		Overall fishery production	OFP	Biomass, abundance, richness, density	Overall fishery production represents the aggregated fishery production (i.e. fish, crab and shrimp) without specifying the species.	Sampling and counting
		Fish production and diversity	FP	Biomass, abundance, richness, density	Mangrove ecosystems are important habitats for diverse fish species.	Sampling and counting
		Crab production and diversity	CP	Biomass, abundance, richness, density, burrow density	Mangrove ecosystems are important habitats for diverse crab species[12].	Sampling and counting
		Shrimp production and diversity	SP	Biomass, abundance, richness, density	Mangrove ecosystems are important habitats for diverse shrimp species.	Sampling and counting
		Other macrobenthic production and diversity	MaP	Biomass, abundance, richness, density	Mangrove ecosystems are important habitats for macrobenthic fauna that forms an important link between the primary detritus at the base of the food web and consumers of higher trophic levels[13]. This includes diverse species of molluscs, gastropods, polychaeta, etc., *excluding* crabs and shrimps (see above).	Collection and treatment of sediment samples
		Microbial diversity	MiP	Richness	Mangrove ecosystems are important habitats for microbial communities that in turn play essential roles in the functioning and maintenance of mangrove ecosystem[14].	Microbial counts
		Soil protozoa diversity	SPr	Abundance, diversity	Mangrove ecosystems are important habitats for diverse species of soil protozoa that: (a) decompose benthic residual deposit, (b) form and develop mangrove soil, and (c) accelerate the mineralization processes for carbon, nitrogen, and other nutrients[15].	Field sampling and laboratory analysis
		Epibiotic diversity	Ep	Biomass, abundance,	Epibiotic communities in mangrove ecosystems play an important role for the growth of mangrove roots[16], the increase of microhabitat complexity[17] and the provision of food to foraging fish[18].	Sampling and counting
		Control of invasive species	CIS	Relative growth rates	Exotic mangrove species can control invasive exotic plants[19].	Plant survey and soil survey
Other functions		Sediment accretion	SA	Surface elevation change	Mangroves retent sediment and accelerate land-building processes in tide-dominated coastal and estuarine environments[20].	Experimental design and measurement
		Wave dissipation	WD	Rate of wave reduction	Coastal mangroves reduce risk from natural hazards by attenuating incident waves, and trapping and stabilizing sediments[21].	Transects design and measurement

Supplementary Table 2. Quality criteria for reviewed studies. Source:[22].

Quality checklist question	Score Yes=1, No=0
INTERNAL VALIDITY	
Research aim	
1. Does the study address a clearly focused question?	
2. Does the question match the answer?	
Data collection	
3. Was the population/area of interest defined in space, time and size?	
4. Selection bias: Was the sample area representative for the population defined?	
5. Was the sample size appropriate?	
6. Was probability/random sampling used for constructing the sample?	
7. If secondary data were used, did an evaluation of the original data take place?	
8. If data collection took place in form of a questionnaire, was it pre-tested/piloted?	
9. Were the data collection methods described in sufficient detail to permit replication?	
Analysis	
10. Were the statistical/analytical methods described in sufficient detail to permit replication?	
11. Is the choice of statistical/analytical methods appropriate and/or justified?	
12. Was uncertainty assessed and reported?	
Results and Conclusions	
13. Do the data support the outcome?	
14. Magnitude of effect: Is the effect large, significant and/or without large uncertainty?	
15. Are all variables and statistical measures reported?	
16. Attrition bias: Are non-response/drop-outs given and is their impact discussed?	
DESIGN-SPECIFIC ASPECTS	
Review	
17. Is there a low probability of publication bias?	
18. Is the review based on several strong-evidence individual studies?	
19. Do the studies included respond to the same question?	
20. Are results between individual studies consistent and homogeneous?	
21. Was the literature searched in a systematic and comprehensive way?	
22. Was a meta-analysis included?	
23. Were appropriate a priori study inclusion/exclusion criteria defined?	
24. Did at least two people select studies and extract data?	
Study with a reference/control	
25. Allocation bias: Was the assignment of case-control groups randomized?	
26. Were groups designed equally, aside from the investigated point of interest?	
27. Performance bias: Was the sampling blinded?	
28. Were there sufficient replicates of treatment and reference groups?	
29. Detection bias: Were outcomes equally measured and determined between groups?	
Observational studies	
30. Were confounding factors identified and strategies to deal with them stated?	
FOCUS-SPECIFIC ASPECTS	
Quantification	
31. Is the unit of the quantification measurement appropriate?	
32. Was temporal change (e.g. annual or long-term) of quantities measured (e.g. species abundance or an ecosystem service) discussed?	
Valuation	
33. If discounting of future costs and outcomes is necessary, was it performed correctly?	
34. If aggregate economic values for a population were estimated, was this estimation consistent with the sampling and the definition of the population?	
Management	
35. Was the aim of the management intervention clearly defined?	
36. Were side effects and tradeoffs on other non-target species, ecosystem services or stakeholders considered?	
37. Were both long-term and short-term effects discussed?	
38. Did monitoring take place for an appropriate time period?	
39. Appropriate outcome measures: Are all relevant outcomes measured in a reliable way?	
Governance	
40. Were long-term effects assessed?	
41. Was the policy instrument that was used described?	
42. Was the influence of the applied policy instrument (incentive/law) on the society discussed?	
43. Appropriate outcome measures: Are all relevant outcomes measured in a reliable way?	

Note: Each study receives one point for each criterion answered with a 'yes', else it receives zero points. If a question is not applicable for a given study it may be left out (especially for design-specific and focus-specific aspects). For instance, our database includes only two review studies, for which the review-specific criteria apply (i.e. Criteria 17-24). All scores are added and the final score is expressed as a percentage. Based on the aggregate score studies are characterized as having (a) Weak evidence (score is <24%); (b) Moderate evidence (score is 25%-49%); (c) Strong evidence (score is 50%-74%); and (d) Very strong evidence (score is >75%).

Supplementary Table 3. Count of observations for each study function by country. Refer to Supplementary Table 1 for function abbreviations. Source data are provided as a Source Data file.

	PP	Ph	BP	CS	OM	N	P	S	ON	HM	LD	WT	GHG	GB	AD	OFP	FP	CP	SP	MaP	MiP	SPr	Ep	CIS	SA	WD
Asia	***3***	***2***	***31***	***42***	***9***	***18***	***11***	***4***	***5***	***12***	***1***	***3***	***1***	***2***		***1***	***4***	***11***	***1***	***14***	***4***	***2***		***1***	***2***	***2***
Bangladesh														1												
China		2	7	11	5	5	4	1	2	8	5	3	3					1		9	3	2		1		
India	1		4	7		1	1		1								1	2			1					
Indonesia	2		2	4		3					2		4					1		1						
Malaysia				2	1	3	2	2	1	2										1						
Myanmar			2	1																						
Philippines			6	2	2	2	2						1	1		1	3	4	1	1						
Sri Lanka			1	1		1																			2	
Thailand			1	5		2	1	1	1	2								2		1						
Vietnam			8	9	1	1	1				3		2					1		1						2
North America				***4***	***2***	***4***	***1***						***1***		***1***		***4***	***1***		***2***					***1***	***1***
Bahamas																	1									
Mexico				1		1									1											
Qatar																	1			1						
USA				3	2	3	1						1				2	1		1					1	1
South America		***1***	***3***	***3***		***1***			***2***	***1***			***1***	***2***				***1***		***3***						
Brazil		1	2	2		1			1	1				1				1		3						
Colombia									1				1	1												
Ecuador			1	1																						
Africa			***4***	***3***	***1***	***1***	***1***				***2***						***1***	***1***	***1***				***1***		***1***	
Kenya			4	3							2						1	1	1				1			
Mauritius					1																				1	
South Africa						1	1																			
Oceania						***1***	***1***													***1***						***1***
New Zealand						1	1													1						1

Supplementary Table 4. Count of observations for each function by publication year. Refer to Supplementary Table 1 for function abbreviations. Source data are provided as a Source Data file.

	PP	Ph	BP	CS	OM	N	P	S	ON	HM	LD	WT	GHG	GB	AD	OFP	FP	CP	SP	MaP	MiP	SPr	Ep	CIS	SA	WD
1992			1								1															
1994																	1				1					
1997																										1
1998				1	1	1	1	1		1																
1999																	2		1							
2000					1	1	1				1		1													
2002																		2								
2003						1	1											1			1					
2004			1	2		1	1			1							1	1								
2005			1								2		1						1							
2006																		1					1			
2007																		1			1					
2008		1	1	3	1	1	1															1				
2009			3	1	1	1	1				1	1		1				1				1			1	
2010			2	1		2	2			1															1	
2011			2	1		1											1				2					
2012			3	5	1	2						1									2					
2013		1	4	3	1				2	1	1		1				1									
2014			5	4		2	1	1	1	1	3		1				1	1			3					
2015			2															2			3			1		1
2016			4	5		2				1			1					2			3					
2017			3	6	4	5	4		1	3		1	1	1			1			1	3				1	
2018		1	1	6	2	2		2	3	2			1	2		1	1	1		1						1
2019	3		5	11		3	1			2	1		5		1			1			1				1	1
2020				3							2									2						

Supplementary Table 5. Two-sided Cochran's Q statistic test for different types of restoration outcomes.

	Function	Cochran's Q	df (=n-1)	*P*-value
Restored mangroves vs. Natural mangroves	Primary productivity	2.59	2	0.2739
	Biomass production	8039.92	76	< 0.0001
	Carbon sequestration	1691.18	59	< 0.0001
	Organic matter accumulation	830.39	24	< 0.0001
	Nitrogen accumulation	453.11	32	< 0.0001
	Phosphorus accumulation	985.08	19	< 0.0001
	Sulphur accumulation	6.38	5	0.2713
	Other nutrient accumulation	10662.52	47	< 0.0001
	Heavy metal accumulation	11178.66	88	< 0.0001
	Litter decomposition	899.86	22	< 0.0001
	Wastewater treatment	2.61	5	0.7595
	GHG emissions	370.18	19	< 0.0001
	**Biogeochemical functions**	**43117.46**	**411**	**< 0.0001**
	Genetic diversity	4.50	1	0.0338
	Avian diversity	-	0	-
	Overall fishery production	1921.94	19	< 0.0001
	Fish production and diversity	168.37	11	< 0.0001
	Crab production and diversity	1480.20	48	< 0.0001
	Shrimp production and diversity	39.46	8	< 0.0001
	Other macrobenthic production and diversity	685.45	46	< 0.0001
	Microbial diversity	4.10	10	0.9429
	**Ecological functions**	**9117.00**	**151**	**< 0.0001**
	Wave dissipation	-	0	-
	**Overall restoration outcomes**	**52978.79**	**564**	**< 0.0001**
Restored mangroves vs. Unvegetated tidal flats	Carbon sequestration	67659.84	87	< 0.0001
	Organic matter accumulation	376.28	11	< 0.0001
	Nitrogen accumulation	7432.92	25	< 0.0001
	Phosphorus accumulation	102.20	8	< 0.0001
	Sulphur accumulation	567.14	17	< 0.0001
	Other nutrient accumulation	55425.00	20	< 0.0001
	Heavy metal accumulation	17025.49	23	< 0.0001
	Litter decomposition	37.12	3	< 0.0001
	Wastewater treatment	118.93	7	< 0.0001
	GHG emissions	126.39	14	< 0.0001
	**Biogeochemical functions**	**348295.16**	**224**	**< 0.0001**
	Fish production and diversity	67.60	4	< 0.0001
	Crab production and diversity	52.64	4	< 0.0001
	Other macrobenthic production and diversity	644.21	51	< 0.0001
	Microbial diversity	782.66	8	< 0.0001
	**Ecological functions**	**3014.23**	**70**	**< 0.0001**
	Wave dissipation	-	0	-
	**Overall restoration outcomes**	**351745.82**	**296**	**< 0.0001**
Restored mangroves vs. Naturally-regenerated mangroves	Biomass production	2710.83	12	< 0.0001
	Carbon sequestration	1139.91	10	< 0.0001
	Organic matter accumulation	46.50	3	< 0.0001
	Nitrogen accumulation	-	0	-
	Other nutrient accumulation	66.64	9	< 0.0001
	Heavy metal accumulation	573618297.85	23	< 0.0001
	Litter decomposition	62.57	1	< 0.0001
	GHG emissions	43.65	3	< 0.0001
	**Biogeochemical functions**	**573626899.91**	**68**	**< 0.0001**
	Crab production and diversity	0.76	3	0.8583
	**Ecological functions**	**0.76**	**3**	**0.8583**
	**Overall restoration outcomes**	**573626942.38**	**72**	**< 0.0001**
Restored mangroves vs. Degraded mangroves	Organic matter accumulation	-	0	-
	Other nutrient accumulation	7.68	5	0.1748
	GHG emissions	277.36	3	< 0.0001
	**Biogeochemical functions**	**376.28**	**10**	**< 0.0001**
	Avian diversity	-	0	-
	Crab production and diversity	8.17	5	0.147
	Shrimp production and diversity	35.51	7	< 0.0001
	**Ecological functions**	**111.72**	**13**	**< 0.0001**
	**Overall restoration outcomes**	**941.22**	**25**	**< 0.0001**

Supplementary Table 6. Adjusted estimates after excluding outliers.

	Function	Original estimate			No. of outliers	Adjusted estimate		
		RR'	Low	High		RR'	Low	High
Restored mangroves vs. Natural mangroves	Primary productivity	0.05	-0.07	0.17	0	0.05	-0.07	0.17
	Biomass production	-0.37	-0.76	0.01	1	-0.21	-0.46	0.03
	Carbon sequestration	-0.33	-0.53	-0.14	1	-0.31	-0.48	-0.14
	Organic matter accumulation	-0.53	-1.04	-0.03	0	-0.53	-1.04	-0.03
	Nitrogen accumulation	-0.39	-0.67	-0.11	2	-0.22	-0.32	-0.12
	Phosphorus accumulation	-0.02	-0.21	0.17	1	-0.07	-0.26	0.12
	Sulphur accumulation	0.10	0.01	0.20	0	0.10	0.01	0.20
	Other nutrient accumulation	-0.09	-0.21	0.03	1	-0.13	-0.26	0.01
	Heavy metal accumulation	-0.16	-0.42	0.10	0	-0.16	-0.42	0.10
	Litter decomposition	-0.20	-0.40	-0.01	2	-0.23	-0.47	0.02
	Wastewater treatment	0.19	-0.12	0.50	1	0.33	-0.03	0.68
	GHG emissions	0.10	-0.14	0.34	2	0.12	-0.12	0.35
	**Biogeochemical functions**	**-0.23**	**-0.34**	**-0.12**	**14**	**-0.30**	**-0.45**	**-0.15**
	Genetic diversity	-0.08	-0.10	-0.05	-	-	-	-
	Avian diversity	0.19	-0.12	0.50	-	-	-	-
	Overall fishery production	2.09	2.03	2.14	1	1.05	0.97	1.13
	Fish production and diversity	-0.39	-1.57	0.79	1	0.04	-1.09	1.16
	Crab production and diversity	-0.37	-0.96	0.22	2	-0.38	-1.11	0.34
	Shrimp production and diversity	0.25	-0.74	1.25	1	-0.22	-0.40	-0.03
	Other macrobenthic production and diversity	-0.10	-0.38	0.18	1	-0.05	-0.28	0.17
	Microbial diversity	-0.08	-0.34	0.19	1	-0.01	-0.43	0.40
	**Ecological functions**	**-0.16**	**-0.46**	**0.14**	**6**	**-0.20**	**-0.48**	**0.08**
	Wave dissipation	-0.69	-1.05	-0.34	-	-	-	-
	**Overall restoration outcomes**	**-0.21**	**-0.34**	**-0.08**	**16**	**-0.24**	**-0.38**	**-0.11**
Restored mangroves vs. Unvegetated tidal flats	Carbon sequestration	0.64	0.35	0.94	0	0.64	0.35	0.94
	Organic matter accumulation	0.55	-0.08	1.18	0	0.55	-0.08	1.18
	Nitrogen accumulation	0.45	0.19	0.72	1	0.36	0.15	0.56
	Phosphorus accumulation	0.06	-0.08	0.21	1	0.06	-0.06	0.17
	Sulphur accumulation	0.10	0.09	0.12	1	0.14	0.05	0.24
	Other nutrient accumulation	-0.12	-0.22	-0.01	2	-0.08	-0.24	0.07
	Heavy metal accumulation	0.41	-0.10	0.93	0	0.41	-0.10	0.93
	Litter decomposition	-0.01	-0.09	0.08	0	-0.01	-0.09	0.08
	Wastewater treatment	0.28	0.26	0.29	0	0.28	0.26	0.29
	GHG emissions	0.96	-0.50	2.42	0	0.96	-0.50	2.42
	**Biogeochemical functions**	**0.46**	**0.24**	**0.68**	**0**	**0.46**	**0.24**	**0.68**
	Fish production and diversity	0.33	-0.82	1.48	0	0.33	-0.82	1.48
	Crab production and diversity	1.03	-0.64	2.70	0	1.03	-0.64	2.70
	Other macrobenthic production and diversity	0.05	-0.23	0.33	1	0.09	-0.12	0.30
	Microbial diversity	1.37	1.31	1.44	0	1.37	1.31	1.44
	**Ecological functions**	**0.37**	**-0.06**	**0.80**	**1**	**0.28**	**-0.07**	**0.64**
	Wave dissipation	1.39	0.75	2.02	-	-	-	-
	**Overall restoration outcomes**	**0.43**	**0.23**	**0.63**	**2**	**0.40**	**0.21**	**0.59**
Restored mangroves vs. Naturally-regenerated mangroves	Biomass production	-0.12	-1.18	0.94	0	-0.12	-1.18	0.94
	Carbon sequestration	-0.16	-0.98	0.66	0	-0.16	-0.98	0.66
	Organic matter accumulation	-0.23	-0.80	0.34	1	0.07	-0.08	0.22
	Nitrogen accumulation	0.51	-0.19	1.20	-	-	-	-
	Other nutrient accumulation	-0.33	-0.80	0.14	0	-0.33	-0.80	0.14
	Heavy metal accumulation	-0.57	-0.87	-0.27	2	-0.43	-0.69	-0.18
	Litter decomposition	-0.18	-0.39	0.03	-	-	-	-
	GHG emissions	1.89	1.65	2.12	0	1.89	1.65	2.12
	**Biogeochemical functions**	**-0.33**	**-0.76**	**0.10**	**1**	**-0.17**	**-0.55**	**0.20**
	Crab production and diversity	0.79	0.38	1.20	0	0.79	0.38	1.20
	**Ecological functions**	**0.79**	**0.38**	**1.20**	**0**	**0.79**	**0.38**	**1.20**
	**Overall restoration outcomes**	**-0.33**	**-0.97**	**0.30**	**1**	**-0.17**	**-0.67**	**0.33**
Restored mangroves vs. Degraded mangroves	Organic matter accumulation	-1.09	-1.18	-1.01	-	-	-	-
	Other nutrient accumulation	-0.50	-0.96	-0.04	1	-0.30	-0.68	0.08
	GHG emissions	0.19	-0.07	0.46	0	0.19	-0.07	0.46
	**Biogeochemical functions**	**-0.94**	**-1.02**	**-0.86**	**1**	**-0.90**	**-0.98**	**-0.82**
	Avian diversity	-0.04	-0.30	0.22	-	-	-	-
	Crab production and diversity	0.80	0.64	0.97	0	0.80	0.64	0.97
	Shrimp production and diversity	0.18	-0.53	0.88	3	0.69	-0.21	1.59
	**Ecological functions**	**0.23**	**-0.33**	**0.79**	**0**	**0.23**	**-0.33**	**0.79**
	**Overall restoration outcomes**	**-0.07**	**-0.77**	**0.63**	**1**	**0.10**	**-0.38**	**0.57**

Note: Outliers are identified using Cook's Distance (see Supplementary Fig. 10).

Supplementary Table 7. Characteristics of mangrove forests in the reviewed economic studies.

Source	Economic variables	Species	Stand age	Restoration method	Location
Chow, 2015	Benefit	-	-	Plantation	Bangladesh
Das, 2017	Benefit	*A. marina*	7	Plantation	India
de Rezende et al., 2015	Benefit	-	20	Plantation	Brazil
Gevaña et al., 2017	Benefit	*R. stylosa*	20	Plantation	Philippines
Lahjie et al., 2019	Benefit		20	Plantation	Indonesia
Pham et al., 2018	Benefit	-	-	-	Vietnam
Rao, 2009	Benefit	-	-	Plantation	India
Russell and Greening, 2013	Benefit	-	18	-	United States
Saenger and Siddiqi, 1993	Benefit	-	-	Plantation	Bangladesh
Susilo et al., 2018	Benefit	-	-	-	Indonesia
Tan et al., 2018	Benefit	-	-	-	China
Tri et al., 1998	Benefit	-		Plantation	Vietnam
Triño and Rodriguez, 2002	Benefit	*Rhizophora spp.*	-	Plantation	Philippines
Tuan et al., 2014	Benefit	-	-	-	Vietnam
Webb and Than, 2000	Benefit	*A. officinalis*	15	Plantation	Myanmar
Brown et al., 2014	Cost	-		Plantation and hydrological rehabilitation	Indonesia
Chowdhury et al., 2019	Cost	*A. marina*	5	Direct propagule dibbling method	India
		R. mucronata	5	Drain and trench method	
		X. moluccensis	5	Transplantation of nursery raised seedlings	
		C. tagal	5	Transplantation of nursery raised seedlings	
		B. sexangula	5	Transplantation of nursery raised seedlings	
Gilman and Ellison, 2007	Cost	*R. mangle*	0.5	Plantation	United States
Hashim et al., 2010	Cost	-	-	Plantation	Malaysia
Motamedi et al., 2014	Cost	*R. apiculata*	6	Hydrological rehabilitation	Malaysia
		A. marina	6	Plantation	
Primavera and Esteban, 2008	Cost	-	-	Plantation	Philippine
Cameron et al., 2019	Benefit and cost	-	10	Plantation and hydrological rehabilitation	Indonesia
Kairo et al., 2009	Benefit and cost	*R. mucronata*	12	Plantation	Kenya
Rahman and Mahmud, 2018	Benefit and cost	-	-	Plantation	Bangladesh
Stone et al., 2008	Benefit and cost	-	4	Plantation	India
Susilo et al., 2017	Benefit and cost	-	5	Plantation	Indonesia
		-	10	Plantation	
Susilo et al., 2017	Benefit and cost	-	-	-	Indonesia
Walton et al., 2006	Benefit and cost	*Rhizophora spp.*	15	Plantation	Philippine

Note: "-" indicates missing data.

Supplementary Table 8. Sensitivity analysis for different social discount rates.

Discount rate	Benefit-cost ratio		
	Restored mangroves [a]	Restored mangroves [b]	Natural mangroves
-2%	10.50	3.36	16.75
4.5%	7.92	2.54	12.64
8%	6.83	2.19	10.90

Note: (a) uses estimated values for each ecosystem services; (b) uses the mean value of the estimated total benefit.

List of publications used in this synthesis.

1. Al-Khayat, J. A. & Jones, D. A. A comparison of the macrofauna of natural and replanted mangroves in Qatar. *Estuar. Coast. SHELF Sci.* **49**, 55–63 (1999).
2. Al-Khayat, J. A., Abdulla, M. A. & Alatalo, J. M. Diversity of benthic macrofauna and physical parameters of sediments in natural mangroves and in afforested mangroves three decades after compensatory planting. *Aquat. Sci.* **81**, 1–11 (2018).
3. Alongi, D. M., Tirendi, F., Trott, L. A. & Xuan, T. T. Benthic decomposition rates and pathways in plantations of the mangrove Rhizophora apiculata in the Mekong delta, Vietnam. *Mar. Ecol. Prog. Ser.* **194**, 87–101 (2000).
4. Alongi, D. M. *et al.* Rapid sediment accumulation and microbial mineralization in forests of the mangrove Kandelia candel in the Jiulongjiang Estuary, China. *Estuar. Coast. Shelf Sci.* **63**, 605–618 (2005).
5. Alongi, D. M., Sasekumar, A., Tirendi, F. & Dixon, P. The influence of stand age on benthic decomposition and recycling of organic matter in managed mangrove forests of Malaysia. *J. Exp. Mar. Bio. Ecol.* **225**, 197–218 (1998).
6. Alongi, D. M., Wattayakorn, G., Tirendi, F. & Dixon, P. Nutrient capital in different aged forests of the mangrove Rhizophora apiculata. *Bot. Mar.* **47**, 116–124 (2004).
7. Ariyanto, D., Bengen, D. G., Prartono, T. & Wardiatno, Y. The physicochemical factors and litter dynamics (Rhizophora mucronata lam. and Rhizophora stylosa griff) of replanted Mangroves, Rembang, Central Java, Indonesia. *Environ. Nat. Resour. J.* **17**, 11–19 (2019).
8. Asadi, M. A., Yona, D. & Fikri, M. Z. Comparing carbon in sediment of primary and artificially generated mangrove forests. *Disaster Adv.* **11**, 18–26 (2018).
9. Ashton, E. C., Hogarth, P. J. & MacIntosh, D. J. A comparison of brachyuran crab community structure at four mangrove locations under different management systems along the Melaka Straits-Andaman Sea Coast of Malaysia and Thailand. *ESTUARIES* **26**, 1461–1471 (2003).
10. Aung, T. T., Than, M. M., Katsuhiro, O. & Yukira, M. Assessing the status of three mangrove species restored by the local community in the cyclone-affected area of the Ayeyarwady Delta, Myanmar. *Wetl. Ecol. Manag.* **19**, 195–208 (2011).
11. Bandibas, M. B. & Hilomen, V. V. Crab biodiversity under different management schemes of mangrove ecosystems. *Glob. J. Environ. Sci. Manag.* **2**, 19–30 (2016).
12. Bao, T. Q. Effect of mangrove forest structures on wave attenuation in coastal Vietnam. *Oceanologia* **53**, 807–818 (2011).
13. Barnuevo, A., Asaeda, T., Sanjaya, K., Kanesaka, Y. & Fortes, M. Drawbacks of mangrove rehabilitation schemes: Lessons learned from the large-scale mangrove plantations. *Estuar. Coast. Shelf Sci.* **198**, 432–437 (2017).
14. Bosire, J. O., Dahdouh-Guebas, F., Kairo, J. G., Cannicci, S. & Koedam, N. Spatial variations in macrobenthic fauna recolonisation in a tropical mangrove bay. *Biodivers. Conserv.* **13**, 1059–1074 (2004).
15. Bosire, J. O. *et al.* Litter degradation and CN dynamics in reforested mangrove plantations at Gazi Bay, Kenya. *Biol. Conserv.* **126**, 287–295 (2005).
16. Brown, B., Fadillah, R., Nurdin, Y., Soulsby, I. & Ahmad, R. Community Based Ecological Mangrove Rehabilitation (CBEMR) in Indonesia. *S.a.p.i.en.s* **7**, 1–13 (2014).
17. Camacho, L. D. *et al.* Tree biomass and carbon stock of a community-managed mangrove forest in Bohol, Philippines. *Forest Sci. Technol.* **7**, 161–167 (2011).
18. Cameron, C., Hutley, L. B. & Friess, D. A. Estimating the full greenhouse gas emissions offset potential and profile between rehabilitating and established mangroves. *Sci. Total Environ.* **665**, 419–431 (2019).
19. Cameron, C., Hutley, L. B., Friess, D. A. & Brown, B. High greenhouse gas emissions mitigation benefits from mangrove rehabilitation in Sulawesi, Indonesia. *Ecosyst. Serv.* **40**, 101035 (2019).

20. Cameron, C., Hutley, L. B., Friess, D. A. & Brown, B. Community structure dynamics and carbon stock change of rehabilitated mangrove forests in Sulawesi, Indonesia. *Ecol. Appl.* **29**, (2019).
21. Cameron, C., Hutley, L. B., Friess, D. A. & Munksgaard, N. C. Hydroperiod, soil moisture and bioturbation are critical drivers of greenhouse gas fluxes and vary as a function of landuse change in mangroves of Sulawesi, Indonesia. *Sci. Total Environ.* **654**, 365–377 (2019).
22. Canales-Delgadillo, J. C. *et al.* The effect of mangrove restoration on avian assemblages of a coastal lagoon in southern Mexico. *PeerJ* **2019**, 1–26 (2019).
23. Chen, G. C. & Ye, Y. Restoration of Aegiceras corniculatum mangroves in Jiulongjiang Estuary changed macro-benthic faunal community. *Ecol. Eng.* **37**, 224–228 (2011).
24. Chen, G. *et al.* Soil greenhouse gas emissions reduce the contribution of mangrove plants to the atmospheric cooling effect. *Environ. Res. Lett.* **11**, (2016).
25. Chen, G., Gao, M., Pang, B., Chen, S. & Ye, Y. Top-meter soil organic carbon stocks and sources in restored mangrove forests of different ages. *For. Ecol. Manage.* **422**, 87–94 (2018).
26. Chen, G.-C., Ye, Y. & Lu, C.-Y. Changes of macro-benthic faunal community with stand age of rehabilitated Kandelia candel mangrove in Jiulongjiang Estuary, China. *Ecol. Eng.* **31**, 215–224 (2007).
27. Chen, H. *et al.* Effects of planting patterns on heavy metals (Cd, As) in soils following mangrove wetlands restoration. *Int. J. Phytoremediation* **21**, 725–732 (2019).
28. Chen, J., Chen, G., Gu, Y., Zhu, H. & Ye, Y. Fate of leaf litter in restored Kandelia obovata (S. L.) mangrove forests with different ages in Jiulong River Estuary, China. *Restor. Ecol.* **28**, 369–377 (2020).
29. Chen, L. *et al.* Comparison of ecophysiological characteristics between introduced and indigenous mangrove species in China. *Estuar. Coast. Shelf Sci.* **79**, 644–652 (2008).
30. Chen, L., Yan, T., Xiong, Y., Zhang, Y. & Lin, G. Food sources of dominant macrozoobenthos between native and non-native mangrove forests: A comparative study. *Estuar. Coast. SHELF Sci.* **187**, 160–167 (2017).
31. Chen, L., Zan, Q., Li, M., Shen, J. & Liao, W. Litter dynamics and forest structure of the introduced Sonneratia caseolaris mangrove forest in Shenzhen, China. *Estuar. Coast. Shelf Sci.* **85**, 241–246 (2009).
32. Chen, L. *et al.* Comparing carbon sequestration and stand structure of monoculture and mixed mangrove plantations of Sonneratia caseolaris and S. apetala in Southern China. *For. Ecol. Manage.* **284**, 222–229 (2012).
33. Chen, Q.-H., Tam, N. F.-Y., Shin, P. K. S., Cheung, S.-G. & Xu, R.-L. Ciliate communities in a constructed mangrove wetland for wastewater treatment. *Mar. Pollut. Bull.* **58**, 711–719 (2009).
34. Chen, Q.-H., Xu, R.-L., Tam, N. F. Y., Cheung, S. G. & Shin, P. K. S. Use of ciliates (Protozoa : Ciliophora) as bioindicator to assess sediment quality of two constructed mangrove sewage treatment belts in Southern China. *Mar. Pollut. Bull.* **57**, 689–694 (2008).
35. Choosak, S., Thongjoo, C. & Chaichana, R. ECOLOGICAL ROLES OF COMMERCIAL MANGROVE PLANTATION FORESTS FOR BENTHIC MACROINVERTEBRATE COMMUNITIES IN THAILAND. *Appl. Ecol. Environ. Res.* **14**, 421–432 (2016).
36. Chow, J. Spatially Explicit Evaluation of Local Extractive Benefits from Mangrove Plantations in Bangladesh. *J. Sustain. For.* **34**, 651–681 (2015).
37. Chowdhury, A., Naz, A., Bhattacharyya, S. & Sanyal, P. Cost-benefit analysis of `Blue Carbon' sequestration by plantation of few key mangrove species at Sundarban Biosphere Reserve, India. *CARBON Manag.* **9**, 575–586 (2018).
38. Clough, B., Tan, D. T., Phuong, D. X. & Buu, D. C. Canopy leaf area index and litter fall in stands of the mangrove Rhizophora apiculata of different age in the Mekong Delta, Vietnam. *Aquat. Bot.* **66**, 311–320 (2000).

39. Crona, B. I., Holmgren, S. & Rönnbäck, P. Re-establishment of epibiotic communities in reforested mangroves of Gazi Bay, Kenya. *Wetl. Ecol. Manag.* **14**, 527–538 (2006).
40. Crona, B. I., Rönnbäck, P. & Ronnback, P. Use of replanted mangroves as nursery grounds by shrimp communities in Gazi Bay, Kenya. *Estuar. Coast. SHELF Sci.* **65**, 535–544 (2005).
41. Cuc, N. T. K. *et al.* Belowground carbon accumulation in young Kandelia candel (L .) Blanco plantations in Thai Binh River Mouth. *Int. J. Ecol. Dev.* **12**, 107–117 (2009).
42. Cuc, N. T. K., Suzuki, T., van Steveninck, E. D. de R. & Hai, H. Modelling the Impacts of Mangrove Vegetation Structure on Wave Dissipation in Ben Tre Province, Vietnam, under Different Climate Change Scenarios. *J. Coast. Res.* **31**, 340–347 (2015).
43. Cuc, N. T. K. & van Steveninck, E. D. de R. Production Function of Planted Mangroves in Thanh Phu Nature Reserve, Mekong Delta, Vietnam. *J. Coast. Res.* **31**, 1084–1090 (2015).
44. Das, S. Ecological Restoration and Livelihood: Contribution of Planted Mangroves as Nursery and Habitat for Artisanal and Commercial Fishery. *World Dev.* **94**, 492–502 (2017).
45. de Rezende, C. E., Kahn, J. R., Passareli, L. & Vásquez, W. F. An economic valuation of mangrove restoration in Brazil. *Ecol. Econ.* **120,** 296-302 (2015).
46. DelVecchia, A. G. *et al.* Organic carbon inventories in natural and restored Ecuadorian mangrove forests. *PeerJ* **2**, (2014).
47. Deng, J. *et al.* Effects of Wetland Restoration on Sulfur and Arylsulfatase in Mangrove Surface Soils at Jinjiang Estuary (Fujian, China). *WETLANDS* **39**, 393–402 (2019).
48. Deng, J. *et al.* An evaluation on the bioavailability of heavy metals in the sediments from a restored mangrove forest in the Jinjiang Estuary, Fujian, China. *Ecotoxicol. Environ. Saf.* **180**, 501-508, (2019).
49. Dou, Z. *et al.* Hyperspectral Estimation of the Chlorophyll Content in Short-Term and Long-Term Restorations of Mangrove in Quanzhou Bay Estuary, China. *SUSTAINABILITY* **10**, (2018).
50. Duncan, C. *et al.* Rehabilitating mangrove ecosystem services: A case study on the relative benefits of abandoned pond reversion from Panay Island, Philippines. *Mar. Pollut. Bull.* **109**, 772–782 (2016).
51. Dung, L. V., Tue, N. T., Nhuan, M. T. & Omori, K. Carbon storage in a restored mangrove forest in Can Gio Mangrove Forest Park, Mekong Delta, Vietnam. *For. Ecol. Manage.* **380**, 31–40 (2016).
52. Elwin, A., Bukoski, J. J., Jintana, V., Robinson, E. J. Z. & Clark, J. M. Preservation and recovery of mangrove ecosystem carbon stocks in abandoned shrimp ponds. *Sci. Rep.* **9**, (2019).
53. Feng, J. *et al.* Effects of exotic and native mangrove forests plantation on soil organic carbon, nitrogen, and phosphorus contents and pools in Leizhou, China. *Catena* **180**, 1–7 (2019).
54. Feng, J. *et al.* Changes in the Community Structure and Diet of Benthic Macrofauna in Invasive Spartina alterniflora Wetlands Following Restoration with Native Mangroves. *WETLANDS* **34**, 673–683 (2014).
55. Feng, J., Huang, Q., Chen, H., Guo, J. & Lin, G. Restoration of native mangrove wetlands can reverse diet shifts of benthic macrofauna caused by invasive cordgrass. *J. Appl. Ecol.* **55**, 905–916 (2018).
56. Feng, J. *et al.* Effects of short-term invasion of Spartina alterniflora and the subsequent restoration of native mangroves on the soil organic carbon, nitrogen and phosphorus stock. *Chemosphere* **184**, 774–783 (2017).
57. Feng, J., Zhu, X., Wu, H., Ning, C. & Lin, G. Distribution and ecological risk assessment of heavy metals in surface sediments of a typical restored mangrove–aquaculture wetland in Shenzhen, China. *Mar. Pollut. Bull.* **124**, 1033–1039 (2017).
58. Ferreira, A. C., Arruda Bezerra, L. E. & Matthews-Cascon, H. Aboveground carbon stock in a restored neotropical mangrove: influence of management and brachyuran crab assemblage. *Wetl. Ecol. Manag.* **27**, 223–242 (2019).

59. Ferreira, A. C., Ganade, G. & de Attayde, J. L. Restoration versus natural regeneration in a neotropical mangrove: Effects on plant biomass and crab communities. *Ocean Coast. Manag.* **110**, 38–45 (2015).
60. Gevaña, D. T., Camacho, L. D. & Camacho, S. C. Stand density management and blue carbon stock of monospecific mangrove plantation in Bohol, Philippines. *For. Stud.* **66**, 75–83 (2017).
61. Gilman, E. & Ellison, J. Efficacy of alternative low-cost approaches to mangrove restoration, American Samoa. *ESTUARIES AND COASTS* **30**, 641–651 (2007).
62. Gnanamoorthy, P. *et al.* Soil organic carbon stock in natural and restored mangrove forests in Pichavaram south-east coast of India. *INDIAN J. GEO-MARINE Sci.* **48**, 801–808 (2019).
63. Gorman, D. & Turra, A. The role of mangrove revegetation as a means of restoring macrofaunal communities along degraded coasts. *Sci. Total Environ.* **566**, 223–229 (2016).
64. Granado, R., Pinto Neta, L. C., Nunes-Freitas, A. F., Voloch, C. M. & Lira, C. F. Assessing Genetic Diversity after Mangrove Restoration in Brazil: Why Is It So Important? *DIVERSITY-BASEL* **10**, (2018).
65. Guo, P., Sun, Y., Su, H., Wang, M. & Zhang, Y. Spatial and temporal trends in total organic carbon (TOC), black carbon (BC), and toted nitrogen (TN) and their relationships under different planting patterns in a restored coastal mangrove wetland: case study in Fujian, China. *Chem. Speciat. Bioavailab.* **30**, 47–56 (2018).
66. Ha, T. H. *et al.* Belowground carbon sequestration in mature planted mangroves (Northern Viet Nam). *For. Ecol. Manage.* **407**, 191–199 (2018).
67. Hasan, S., Triest, L., Afrose, S. & De Ryck, D. J. R. Migrant pool model of dispersal explains strong connectivity of Avicennia officinalis within Sundarban mangrove areas: Effect of fragmentation and replantation. *Estuar. Coast. SHELF Sci.* **214**, 38–47 (2018).
68. Hashim, R., Kamali, B., Tamin, N. M. & Zakaria, R. An integrated approach to coastal rehabilitation: Mangrove restoration in Sungai Haji Dorani, Malaysia. *Estuar. Coast. SHELF Sci.* **86**, 118–124 (2010).
69. He, Z. *et al.* Appearance can be deceptive: shrubby native mangrove species contributes more to soil carbon sequestration than fast-growing exotic species. *Plant Soil* **432**, 425–436 (2018).
70. Hien, H. T., Marchand, C., Aime, J. & Cuc, N. T. K. Seasonal variability of CO2 emissions from sediments in planted mangroves (Northern Viet Nam). *Estuar. Coast. SHELF Sci.* **213**, 28–39 (2018).
71. Hieu, P. Van, Dung, L. V., Tue, N. T. & Omori, K. Will restored mangrove forests enhance sediment organic carbon and ecosystem carbon storage? *Reg. Stud. Mar. Sci.* **14**, 43–52 (2017).
72. Hong Tinh, P. *et al.* A Comparison of Soil Carbon Stocks of Intact and Restored Mangrove Forests in Northern Vietnam. *Forests* **11**, 660 (2020).
73. Hookham, B., Shau-hwai, A. T., Dayrat, B. & Hintz, W. A Baseline Measure of Tree and Gastropod Biodiversity in Replanted and Natural Mangrove Stands in Malaysia: Langkawi Island and Sungai Merbok. **25**, 1–12 (2014).
74. Hu, C., Shui, B., Li, W., Yang, X. & Zhang, X. Assessing the ecological quality status of transplanted mangrove wetland in the Oujiang estuary, China. *Mar. Pollut. Bull.* **133**, 1–8 (2018).
75. Huxham, M., Kimani, E. & Augley, J. Mangrove fish: A comparison of community structure between forested and cleared habitats. *Estuar. Coast. Shelf Sci.* **60**, 637–647 (2004).
76. Irma, D. & Sofyatuddin, K. Diversity of Gastropods and Bivalves in mangrove ecosystem rehabilitation areas in Aceh Besar and Banda Aceh districts, Indonesia. *AACL Bioflux* **5**, 55–59 (2012).
77. Jin, L., Lu, C. Y., Ye, Y. & Ye, G. F. Soil Respiration in a Subtropical Mangrove Wetland in the Jiulong River Estuary, China. *Pedosphere* **23**, 678–685 (2013).

78. Kairo, J. G., Bosire, J., Langat, J., Kirui, B. & Koedam, N. Allometry and biomass distribution in replanted mangrove plantations at Gazi Bay, Kenya. in *Aquatic Conservation: Marine and Freshwater Ecosystems* (2009). doi:10.1002/aqc.1046
79. Kairo, J. G., Wanjiru, C. & Ochiewo, J. Net pay: Economic analysis of a replanted mangrove plantation in Kenya. *J. Sustain. For.* (2009). doi:10.1080/10549810902791523
80. Kairo, J. G., Lang'at, J. K. S., Dahdouh-Guebas, F., Bosire, J. & Karachi, M. Structural development and productivity of replanted mangrove plantations in Kenya. *For. Ecol. Manage.* **255**, 2670–2677 (2008).
81. Kathiresan, K., Anburaj, R., Gomathi, V. & Saravanakumar, K. Carbon sequestration potential of Rhizophora mucronata and Avicennia marina as influenced by age, season, growth and sediment characteristics in southeast coast of India. *J. Coast. Conserv.* **17**, 397–408 (2013).
82. Kathiresan, K., Gomathi, V., Anburaj, R. & Saravanakumar, K. Impact of mangrove vegetation on seasonal carbon burial and other sediment characteristics in the Vellar-Coleroon estuary, India. *J. For. Res.* **25**, 787–794 (2014).
83. Kathiresan, K., Saravanakumar, K., Anburaj, R. & Gomathi, V. A simple method for assessing mangrove forest based on young plants and sesarmid crab holes. *Reg. Stud. Mar. Sci.* **7**, 204–210 (2016).
84. Kibler, K. M., Kitsikoudis, V., Donnelly, M., Spiering, D. W. & Walters, L. Flow-Vegetation Interaction in a Living Shoreline Restoration and Potential Effect to Mangrove Recruitment. *SUSTAINABILITY* **11**, (2019).
85. Kirui, B. Y. K., Huxham, M., Kairo, J. & Skov, M. Influence of species richness and environmental context on early survival of replanted mangroves at Gazi bay, Kenya. *Hydrobiologia* **603**, 171–181 (2008).
86. Kirui, B. Y. K., Kairo, J. G., Skov, M. W., Mencuccini, M. & Huxham, M. Effects of species richness, identity and environmental variables on growth in planted mangroves in Kenya. *Mar. Ecol. Prog. Ser.* **465**, 1–10 (2012).
87. Konnerup, D., Mauricio Betancourt-Portela, J., Villamil, C. & Pablo Parra, J. Nitrous oxide and methane emissions from the restored mangrove ecosystem of the Cienaga Grande de Santa Marta, Colombia. *Estuar. Coast. SHELF Sci.* **140**, 43–51 (2014).
88. Kridiborworn, P., Chidthaisong, A., Yuttitham, M. & Tripetchkul, S. Carbon Sequestration by Mangrove Forest Planted Specifically for Charcoal Production in Yeesarn, Samut Songkram. *J. Sustain. Energy Environ.* **3**, 87–92 (2012).
89. Kumara, A. M. P., Jayatissa, L. P., Krauss, K. W., Phillips, D. H. & Huxham, M. International Association for Ecology High mangrove density enhances surface accretion , surface elevation change , and tree survival in coastal areas susceptible to sea-level rise Published by : Springer in cooperation with International Association for . **164**, 545–553 (2016).
90. Lahjie, A. M., Nouval, B., Lahjie, A. A., Ruslim, Y. & Kristiningrum, R. Economic valuation from direct use of mangrove forest restoration in Balikpapan Bay, East Kalimantan, Indonesia[version 2; peer review: 2 approved]. *F1000Research* **8**, 1–13 (2019).
91. Lang'at, J. K. S. *et al.* Species mixing boosts root yield in mangrove trees. *Oecologia* **172**, 271–278 (2013).
92. Leung, J. Y. S. Habitat heterogeneity affects ecological functions of macrobenthic communities in a mangrove: Implication for the impact of restoration and afforestation. *Glob. Ecol. Conserv.* **4**, 423–433 (2015).
93. Leung, J. Y. S. S. & Cheung, N. K. M. M. Can mangrove plantation enhance the functional diversity of macrobenthic community in polluted mangroves? *Mar. Pollut. Bull.* **116**, 454–461 (2017).
94. Leung, J. Y. S. & Tam, N. F. Y. Influence of plantation of an exotic mangrove species, Sonneratia caseolaris (L.) Engl., on macrobenthic infaunal community in Futian Mangrove National Nature Reserve, China. *J. Exp. Mar. Bio. Ecol.* **448**, 1–9 (2013).

95. Li, T. & Ye, Y. Dynamics of decomposition and nutrient release of leaf litter in Kandelia obovata mangrove forests with different ages in Jiulongjiang Estuary, China. *Ecol. Eng.* **73**, 454–460 (2014).
96. Li, W. *et al.* Effect of mangrove restoration on crab burrow density in Luoyangjiang Estuary, China. *For. Ecosyst.* **2**, (2015).
97. Li, Y.-F., Du, F.-Y., Gu, Y.-G., Ning, J.-J. & Wang, L.-G. Changes of the Macrobenthic Faunal Community with Stand Age of a Non-native Mangrove Species in Futian Mangrove National Nature Reserve, Guangdong, China. *Zool. Stud.* **56**, (2017).
98. Liao, Q. Y. *et al.* An ecological analysis of soil sarcodina at Dongzhaigang mangrove in Hainan Island, China. *Eur. J. Soil Biol.* **45**, 214–219 (2009).
99. Liu, L. *et al.* Long-term differences in annual litter production between alien (Sonneratia apetala) and native (Kandelia obovata) mangrove species in Futian, Shenzhen, China. *Mar. Pollut. Bull.* **85**, 747–753 (2014).
100. Lu, W. *et al.* Changes in Carbon Pool and Stand Structure of a Native Subtropical Mangrove Forest after Inter-Planting with Exotic Species Sonneratia apetala. *PLoS One* **9**, (2014).
101. Lunstrum, A. & Chen, L. Soil carbon stocks and accumulation in young mangrove forests. *SOIL Biol. Biochem.* **75**, 223–232 (2014).
102. Luo, Z., Sun, O. J. & Xu, H. A comparison of species composition and stand structure between planted and natural mangrove forests in Shenzhen Bay, South China. *J. PLANT Ecol.* **3**, 165–174 (2010).
103. Macintosh, D. J., Ashton, E. C. & Havanon, S. Mangrove rehabilitation and intertidal biodiversity: A study in the Ranong mangrove ecosystem, Thailand. *Estuar. Coast. SHELF Sci.* **55**, 331–345 (2002).
104. Manna, S. *et al.* Estimating aboveground biomass in Avicennia marina plantation in Indian Sundarbans using high-resolution satellite data. *J. Appl. Remote Sens.* **8**, (2014).
105. Matsui, N., Suekuni, J., Nogami, M., Havanond, S. & Salikul, P. Mangrove rehabilitation dynamics and soil organic carbon changes as a result of full hydraulic restoration and re-grading of a previously intensively managed shrimp pond. *Wetl. Ecol. Manag.* **18**, 233–242 (2010).
106. Matsui, N., Morimune, K., Meepol, W. & Chukwamdee, J. Ten Year Evaluation of Carbon Stock in Mangrove Plantation Reforested from an Abandoned Shrimp Pond. *FORESTS* **3**, 431–444 (2012).
107. McKee, K. L. & Faulkner, P. L. Restoration of biogeochemical function in mangrove forests. *Restor. Ecol.* **8**, 247–259 (2000).
108. Montgomery, J. M., Bryan, K. R., Horstman, E. M. & Mullarney, J. C. Attenuation of Tides and Surges by Mangroves: Contrasting Case Studies from New Zealand. *WATER* **10**, (2018).
109. Morrisey, D. J. *et al.* Differences in benthic fauna and sediment among mangrove (Avicennia marina var. australasica) stands of different ages in New Zealand. *Estuar. Coast. SHELF Sci.* **56**, 581–592 (2003).
110. Motamedi, S., Hashim, R., Zakaria, R., Song, K.-I. & Sofawi, B. Long-Term Assessment of an Innovative Mangrove Rehabilitation Project: Case Study on Carey Island, Malaysia. *Sci. WORLD J.* **214**, 953830 (2014).
111. Naidoo, G. Differential effects of nitrogen and phosphorus enrichment on growth of dwarf Avicennia marina mangroves. *Aquat. Bot.* **90**, 184–190 (2009).
112. Nam, V. N., Sasmito, S. D., Murdiyarso, D., Purbopuspito, J. & MacKenzie, R. A. Carbon stocks in artificially and naturally regenerated mangrove ecosystems in the Mekong Delta. *Wetl. Ecol. Manag.* **24**, 231–244 (2016).
113. Nga, B. T., Tinh, H. Q., Tam, D. T., Scheffer, M. & Roijackers, R. Young mangrove stands produce a large and high quality litter input to aquatic systems. *Wetl. Ecol. Manag.* **13**, 569–576 (2005).

114. NGUYEN, H. T. *et al.* The effects of stand-age and inundation on carbon accumulation in mangrove plantation soil in Namdinh, Northern Vietnam. *Tropics* **14**, 21–37 (2004).
115. Okimoto, Y., Nose, A., Oshima, K., Tateda, Y. & Ishii, T. A case study for an estimation of carbon fixation capacity in the mangrove plantation of Rhizophora apiculata trees in Trat, Thailand. *For. Ecol. Manage.* **310**, 1016–1026 (2013).
116. Osland, M. J. *et al.* Ecosystem Development After Mangrove Wetland Creation: Plant-Soil Change Across a 20-Year Chronosequence. *ECOSYSTEMS* **15**, 848–866 (2012).
117. Oxmann, J. F., Pham, Q. H., Schwendenmann, L., Stellman, J. M. & Lara, R. J. Mangrove reforestation in Vietnam: the effect of sediment physicochemical properties on nutrient cycling. *Plant Soil* **326**, 225–241 (2010).
118. Pagliosa, P. R., Oortman, M. S., Rovai, A. S. & Soriano-Sierra, E. J. Is mangrove planting insufficient for benthic macrofaunal recovery when environmental stress is persistent? *Ecol. Eng.* **95**, 290–301 (2016).
119. Perry, C. T. & Berkeley, A. Intertidal substrate modification as a result of mangrove planting: Impacts of introduced mangrove species on sediment microfacies characteristics. *Estuar. Coast. SHELF Sci.* **81**, 225–237 (2009).
120. Peters, J. R., Yeager, L. A. & Layman, C. A. Comparison of fish assemblages in restored and natural mangrove habitats along an urban shoreline. *Bull. Mar. Sci.* **91**, 125–139 (2015).
121. Pham, T. D. *et al.* Willingness to pay for mangrove restoration in the context of climate change in the Cat Ba biosphere reserve, Vietnam. *Ocean Coast. Manag.* **163**, 269–277 (2018).
122. Phan, S. M., Nguyen, H. T. T., Nguyen, T. K. & Lovelock, C. Modelling above ground biomass accumulation of mangrove plantations in Vietnam. *For. Ecol. Manage.* **432**, 376–386 (2019).
123. Phillips, D. H., Kumara, M. P., Jayatissa, L. P., Krauss, K. W. & Huxham, M. Impacts of Mangrove Density on Surface Sediment Accretion, Belowground Biomass and Biogeochemistry in Puttalam Lagoon, Sri Lanka. *WETLANDS* **37**, 471–483 (2017).
124. Primavera, J. H. & Esteban, J. M. A. A review of mangrove rehabilitation in the Philippines: Successes, failures and future prospects. *Wetl. Ecol. Manag.* **16**, 345–358 (2008).
125. Rahman, M. M. & Mahmud, M. A. Economic feasibility of mangrove restoration in the Southeastern Coast of Bangladesh. *Ocean Coast. Manag.* **161**, 211–221 (2018).
126. Rao, R. G. Climate change mitigation through reforestation in Godavari mangroves in India. *Int. J. Clim. Chang. Strateg. Manag.* **1**, 340–355 (2009).
127. Ren, H., Chen, H., Li, Z. & Han, W. Biomass accumulation and carbon storage of four different aged Sonneratia apetala plantations in Southern China. *Plant Soil* **327**, 279–291 (2010).
128. Ren, H. *et al.* Restoration of mangrove plantations and colonisation by native species in Leizhou bay, South China. *Ecol. Res.* **23**, 401–407 (2008).
129. Rönnbäck, P., Troell, M., Kautsky, N. & Primavera, J. H. Distribution pattern of shrimps and fish among Avicennia and Rhizophora microhabitats in the Pagbilao mangroves, Philippines. *Estuar. Coast. Shelf Sci.* **48**, 223–234 (1999).
130. Rovai, A. S. *et al.* Photosynthetic performance of restored and natural mangroves under different environmental constraints. *Environ. Pollut.* **181**, 233–241 (2013).
131. Russell, M. & Greening, H. Estimating Benefits in a Recovering Estuary: Tampa Bay, Florida. *Estuaries and Coasts* **38**, 9–18 (2013).
132. Saenger, P. & Siddiqi, N. A. Land from the sea- The mangrove afforestation program of Banglandesh. *Ocean Coast. Manag.* **20**, 23–39 (1993).
133. Sahu, S. C., Kumar, M. & Ravindranath, N. H. Carbon stocks in natural and planted mangrove forests of Mahanadi Mangrove Wetland, East Coast of India. *Curr. Sci.* **110**, 2253–2260 (2016).
134. Sahu, S. K. & Kathiresan, K. The age and species composition of mangrove forest directly influence the net primary productivity and carbon sequestration potential. *Biocatal. Agric. Biotechnol.* **20**, 101235 (2019).

135. Salas-Leiva, D. E., Mayor-Durán, V. M. & Toro-Perea, N. Genetic diversity of black mangrove (Avicennia germinans) in natural and reforested areas of Salamanca Island Parkway, Colombian Caribbean. *Hydrobiologia* **620**, 17–24 (2009).
136. Salmo, S. G. & Duke, N. C. Establishing mollusk colonization and assemblage patterns in planted mangrove stands of different ages in Lingayen Gulf, Philippines. *Wetl. Ecol. Manag.* **18**, 745–754 (2010).
137. Salmo, S. G., Lovelock, C. E. & Duke, N. C. Assessment of vegetation and soil conditions in restored mangroves interrupted by severe tropical typhoon 'Chan-hom' in the Philippines. *Hydrobiologia* **733**, 85–102 (2014).
138. Salmo, S. G., Lovelock, C. & Duke, N. C. Vegetation and soil characteristics as indicators of restoration trajectories in restored mangroves. *Hydrobiologia* **720**, 1–18 (2013).
139. Salmo, S. G., Tibbetts, I. R. & Duke, N. C. Nekton communities as indicators of habitat functionality in Philippine mangrove plantations. *Mar. Freshw. Res.* **69**, 477–485 (2018).
140. Salmo, S. G., Tibbetts, I. & Duke, N. C. Colonization and shift of mollusc assemblages as a restoration indicator in planted mangroves in the Philippines. *Biodivers. Conserv.* **26**, 865–881 (2017).
141. Saravanakumar, K. *et al.* Soil-microbial communities indexing from mangroves rhizosphere and barren sandy habitats. *Physiol. Mol. Plant Pathol.* **104**, 58–68 (2018).
142. Peralta-Milan, S. A. & Salmo III, S. G. Evaluating Patterns of Fish Assemblage Changes from Different-Aged Reforested Mangroves in Lingayen Gulf. J. Environ. Sci. Manag. (2013).
143. Sen, S., Mukherjee, S., Chaudhuri, A. & Homechaudhuri, S. Temporal changes in brachyuran crab diversity along heterogeneous habitat in a mangrove ecosystem of Indian Sundarbans. *Sci. Mar.* **78**, 433–442 (2014).
144. Sharma, S. *et al.* Growth performance and structure of a mangrove afforestation project on a former seagrass bed, Mindanao Island, Philippines. *Hydrobiologia* **803**, 359–371 (2017).
145. Sidik, F., Adame, M. F. & Lovelock, C. E. Carbon sequestration and fluxes of restored mangroves in abandoned aquaculture ponds. *J. INDIAN Ocean Reg.* **15**, 177–192 (2019).
146. Smith, N. F., Wilcox, C. & Lessmann, J. M. Fiddler crab burrowing affects growth and production of the white mangrove (Laguncularia racemosa) in a restored Florida coastal marsh. *Mar. Biol.* **156**, 2255–2266 (2009).
147. Sofawi, A. B., Rozainah, M. Z., Normaniza, O. & Roslan, H. Mangrove rehabilitation on Carey Island, Malaysia: an evaluation of replanting techniques and sediment properties. *Mar. Biol. Res.* **13**, 390–401 (2017).
148. Soper, F. M. *et al.* Non-native mangroves support carbon storage, sediment carbon burial, and accretion of coastal ecosystems. *Glob. Chang. Biol.* **25**, 4315–4326 (2019).
149. Stone, K., Bhat, M., Bhatta, R. & Mathews, A. Factors influencing community participation in mangroves restoration: A contingent valuation analysis. *Ocean Coast. Manag.* **51**, 476–484 (2008).
150. Sukardjo, S. & Yamada, I. Biomass and productivity of a Rhizophora Mucronata Lamarck plantation in Tritih, Central Java, Indonesia. *For. Ecol. Manage.* **49**, 195–209 (1992).
151. SUSILO, H., TAKAHASHI, Y., SATO, G., NOMURA, H. & YABE, M. The Adoption of Silvofishery System to Restore Mangrove Ecosystems and Its Impact on Farmers' Income in Mahakam Delta, Indonesia. *Journal of the Faculty of Agriculture, Kyushu* University **63**, 433–442 (2018).
152. Susilo, H., Takahashi, Y. & Yabe, M. Evidence for Mangrove Restoration in the Mahakam Delta, Indonesia, Based on Households' Willingness to Pay. *J. Agric. Sci.* **9**, 30 (2017).
153. Susilo, H., Takahashi, Y. & Yabe, M. The opportunity cost of labor for valuing mangrove restoration in Mahakam Delta, Indonesia. *Sustain.* **9**, 1–13 (2017).

154. Suzuki, K. N. *et al.* Metal sorption by sediments from a mangrove reforestation area in Guanabara Bay (Se Brazil) Revealed by using radiotracers. *J. Sediment. Environ.* **1**, 292–296 (2016).
155. Tamooh, F. *et al.* Below-ground root yield and distribution in natural and replanted mangrove forests at Gazi bay, Kenya. *For. Ecol. Manage.* **256**, 1290–1297 (2008).
156. Tan, Y. *et al.* Valuation of environmental improvements in coastal wetland restoration: A choice experiment approach. *Glob. Ecol. Conserv.* **15**, e00440 (2018).
157. Tang, Y. *et al.* Ecological influence of exotic plants of Sonneratia apetala on understory macrofauna. *Acta Oceanol. Sin.* **31**, 115–125 (2012).
158. Tang, Y. *et al.* Ecological indicators showing the succession of macrofauna communities in sonneratia apetala artificial mangrove wetlands on Qi'ao Island at Zhuhai, South China. *Acta Oceanol. Sin.* **33**, 62–72 (2014).
159. Thant, Y. M., Kanzaki, M., Ohta, S. & Than, M. M. Carbon sequestration by mangrove plantations and a natural regeneration stand in the Ayeyarwady Delta, Myanmar. *Tropics* **21**, 1–10 (2012).
160. Thongjoo, C., Choosak, S. & Chaichana, R. Soil fertility improvement from commercial monospecific mangrove forests (Rhizophora apiculata) at Yeesarn Village, Samut Songkram Province, Thailand. *Trop. Ecol.* **59**, 91–97 (2018).
161. Thornton, S. R. & Johnstone, R. W. Mangrove rehabilitation in high erosion areas: Assessment using bioindicators. *Estuar. Coast. SHELF Sci.* **165**, 176–184 (2015).
162. Tian, T. *et al.* Performance and bacterial community structure of a 10-years old constructed mangrove wetland. *Mar. Pollut. Bull.* **124**, 1096–1105 (2017).
163. Tian, T. *et al.* Changes of substrate microbial biomass and community composition in a constructed mangrove wetland for municipal wastewater treatment during 10-years operation. *Mar. Pollut. Bull.* **155**, (2020).
164. Tri, N. H., Adger, W. N. & Kelly, P. M. Natural resource management in mitigating climate impacts: the example of mangrove restoration in Vietnam. *Glob. Environ. Chang. POLICY Dimens.* **8**, 49–61 (1998).
165. Triño, A. T. & Rodriguez, E. M. Pen culture of mud crab Scylla serrata in tidal flats reforested with mangrove trees. *Aquaculture* **211**, 125–134 (2002).
166. Tuan, T. H., My, N. H. D., Anh, L. T. Q. & Toan, N. Van. Using contingent valuation method to estimate the WTP for mangrove restoration under the context of climate change: A case study of Thi Nai lagoon, Quy Nhon city, Vietnam. *Ocean Coast. Manag.* **95**, 198–212 (2014).
167. Ulfa, M., Ikejima, K., Poedjirahajoe, E., Faida, L. R. W. & Harahap, M. M. Effects of mangrove rehabilitation on density of Scylla spp. (mud crabs) in Kuala Langsa, Aceh, Indonesia. *Reg. Stud. Mar. Sci.* **24**, 296–302 (2018).
168. Valentine-Rose, L. & Layman, C. A. Response of Fish Assemblage Structure and Function Following Restoration of Two Small Bahamian Tidal Creeks. *Restor. Ecol.* **19**, 205–215 (2011).
169. Vinh, T. Van, Allenbach, M., Linh, K. T. V. & Marchand, C. Changes in Leaf Litter Quality During Its Decomposition in a Tropical Planted Mangrove Forest (Can Gio, Vietnam). *Front. Environ. Sci.* **8**, (2020).
170. Vinh, T. Van, Marchand, C., Linh, T. V. K., Vinh, D. D. & Allenbachd, M. Allometric models to estimate above-ground biomass and carbon stocks in Rhizophora apiculata tropical managed mangrove forests (Southern Viet Nam). *For. Ecol. Manage.* **434**, 131–141 (2019).
171. Vose, F. E. & Bell, S. S. Resident fishes and macrobenthos in mangrove-rimmed habitats – Evaluation of habitat restoration by hydrologic modification. *ESTUARIES* **17**, 585–596 (1994).
172. Vovides, A. G., Bashan, Y., Lopez-Portillo, J. A. & Guevara, R. Nitrogen Fixation in Preserved, Reforested, Naturally Regenerated and Impaired Mangroves as an Indicator of Functional Restoration in Mangroves in an Arid Region of Mexico. *Restor. Ecol.* **19**, 236–244 (2011).

173. Walton, M. E., Le Vay, L., Lebata, J. H., Binas, J. & Primavera, J. H. Seasonal abundance, distribution and recruitment of mud crabs (Scylla spp.) in replanted mangroves. *Estuar. Coast. SHELF Sci.* **66**, 493–500 (2006).
174. Walton, M. E. M., Samonte-Tan, G. P. B., Primavera, J. H., Edwards-Jones, G. & Le Vay, L. Are mangroves worth replanting? The direct economic benefits of a community-based reforestation project. *Environ. Conserv.* **33**, 335–343 (2006).
175. Walton, M. E., Le Vay, L., Lebata, J. H., Binas, J. & Primavera, J. H. Assessment of the effectiveness of mangrove rehabilitation using exploited and non-exploited indicator species. *Biol. Conserv.* **138**, 180–188 (2007).
176. Wang, G., Guan, D., Peart, M. R., Chen, Y. & Peng, Y. Ecosystem carbon stocks of mangrove forest in Yingluo Bay, Guangdong Province of South China. *For. Ecol. Manage.* **310**, 539–546 (2013).
177. Wang'ondu, V. W. *et al.* Litter Fall Dynamics of Restored Mangroves (Rhizophora mucronata Lamk. and Sonneratia alba Sm.) in Kenya. *Restor. Ecol.* **22**, 824–831 (2014).
178. Webb, E. L. & Than, M. M. Optimizing investment strategies for mangrove plantations by considering biological and economic parameters. *J. Coast. Conserv.* **6**, 181–190 (2000).
179. Wiarta, R., Indrayani, Y., Mulia, F. & Astiani, D. Carbon sequestration by young Rhizophora apiculata plants in Kubu Raya district, West Kalimantan, Indonesia. *Biodiversitas* **20**, 311–315 (2019).
180. Wu, M. *et al.* Species choice in mangrove reforestation may influence the quantity and quality of long-term carbon sequestration and storage. *Sci. Total Environ.* **714**, 136742 (2020).
181. Ye, Y., Chen, Y. P. & Chen, G. C. Litter production and litter elemental composition in two rehabilitated Kandelia obovata mangrove forests in Jiulongjiang Estuary, China. *Mar. Environ. Res.* **83**, 63–72 (2013).
182. Yu, C. *et al.* Changes of ecosystem carbon stock following the plantation of exotic mangrove Sonneratia apetala in Qi'ao Island, China. *Sci. Total Environ.* **717**, (2020).
183. Yu, X. *et al.* Sonneratia apetala introduction alters methane cycling microbial communities and increases methane emissions in mangrove ecosystems. *SOIL Biol. Biochem.* **144**, (2020).
184. Zhang, J. P., Shen, C. De, Ren, H., Wang, J. & Han, W. D. Estimating Change in Sedimentary Organic Carbon Content During Mangrove Restoration in Southern China Using Carbon Isotopic Measurements. *Pedosphere* **22**, 58-66 (2012).
185. Zhao, B., Zhou, Y. wu & Chen, G. zhu. The effect of mangrove reforestation on the accumulation of PCBs in sediment from different habitats in Guangdong, China. *Mar. Pollut. Bull.* **64**, 1614–1619 (2012).
186. Zhou, T. *et al.* Use of exotic plants to control Spartina alterniflora invasion and promote mangrove restoration. *Sci. Rep.* **5**, 1–13 (2015).
187. Zhou, Y., Zhao, B., Peng, Y. & Chen, G. Influence of mangrove reforestation on heavy metal accumulation and speciation in intertidal sediments. *Mar. Pollut. Bull.* **60**, 1319–1324 (2010).
188. Zvonareva, S., Kantor, Y., Li, X. & Britayev, T. Long-term monitoring of Gastropoda (Mollusca) fauna in planted mangroves in central Vietnam. *Zool. Stud.* **54**, (2015).

Supplementary References

1. Altman, N. & Krzywinski, M. Analyzing outliers: influential or nuisance? *Nat. Methods* **13**, 281–282 (2016).
2. Field, C. B. *et al.* Mangrove Biodiversity and Ecosystem Function. *Glob. Ecol. Biogeogr. Lett.* **7**, 3 (1998).
3. Twilley, R. R., Castañeda-Moya, E., Rivera-Monroy, V. H. & Rovai, A. Productivity and Carbon Dynamics in Mangrove Wetlands. in *Mangrove Ecosystems: A Global Biogeographic Perspective* (eds. Rivera-Monroy, V. H., Lee, S. Y., Kristensen, E. & Twilley, R. R.) (Springer International Publishing, 2017).
4. Alongi, D. M. Carbon Cycling and Storage in Mangrove Forests. *Annu. Rev. Mar. Sci.* **6**, 195–219 (2014).
5. Alongi, D. M. Effect of mangrove detrital outwelling on nutrient regeneration and oxygen fluxes in coastal sediments of the central Great Barrier Reef lagoon. *Estuar. Coast. Shelf Sci.* **31**, 581–598 (1990).
6. Reef, R., Feller, I. C. & Lovelock, C. E. Nutrition of mangroves. *Tree Physiol.* **30**, 1148–1160 (2010).
7. Kannan, N., Thirunavukkarasu, N., Suresh, A. & Rajagopal, K. Analysis of Heavy Metals Accumulation in Mangroves and Associated mangroves Species of Ennore Mangrove Ecosystem, East Coast India. *Indian J. Sci. Technol.* **9**, (2016).
8. Loría-Naranjo, M. Mangrove Leaf Litter Decomposition in a Seasonal Tropical Environment. *J. Coast. Res.* **35**, 122 (2019).
9. Zhang, J., Liu, J., Ouyang, Y., Liao, B. & Zhao, B. Removal of nutrients and heavy metals from wastewater with mangrove Sonneratia apetala Buch-Ham. *Ecol. Eng.* **36**, 807–812 (2010).
10. Cameron, C., Hutley, L. B. & Friess, D. A. Estimating the full greenhouse gas emissions offset potential and profile between rehabilitating and established mangroves. *Sci. Total Environ.* **665**, 419–431 (2019).
11. Nagelkerken, I. *et al.* The habitat function of mangroves for terrestrial and marine fauna: A review. *Aquat. Bot.* **89**, 155–185 (2008).
12. Jones, D. A. *Crabs of the mangal ecosystem*. (Hydrobiology of the Mangal. W. Junk Publishers, 1984).
13. Macintosh, D. J. *Ecology and Productivity of Malaysisan Mangrove Crab Populations (Decapoda: Brachyura)*. (University of Malaya & UNESCO, 1984).
14. Santana, C. O. *et al. Microbial community structure and ecology in sediments of a pristine mangrove forest*. Preprint at http://biorxiv.org/lookup/doi/10.1101/833814 (2019).
15. Ekelund, F. & RÃ¸nn, R. Notes on protozoa in agricultural soil with emphasis on heterotrophic flagellates and naked amoebae and their ecology. *FEMS Microbiol. Rev.* **15**, 321–353 (1994).
16. Ellison, A. M., Farnsworth, E. J. & Twilley, R. R. Facultative Mutualism Between Red Mangroves and Root-Fouling Sponges in Belizean Mangal. *Ecology* **77**, 2431–2444 (1996).
17. Procheş, Ş., Marshall, D. J., Ugrasen, K. & Ramcharan, A. Mangrove pneumatophore arthropod assemblages and temporal patterns. *J. Mar. Biol. Assoc. U. K.* **81**, 545–552 (2001).
18. Randall, J. E. & Hartman, W. D. Sponge-feeding fishes of the West Indies. *Mar. Biol.* **1**, 216–225 (1968).
19. Zhou, T. *et al.* Use of exotic plants to control Spartina alterniflora invasion and promote mangrove restoration. *Sci. Rep.* **5**, 12980 (2015).
20. Chaudhuri, P., Chaudhuri, S. & Ghosh, R. The Role of Mangroves in Coastal and Estuarine Sedimentary Accretion in Southeast Asia. in *Sedimentation Engineering [Working Title]* (IntechOpen, 2019).
21. Horstman, E. M. *et al.* Wave attenuation in mangroves: A quantitative approach to field observations. *Coast. Eng.* **94**, 47–62 (2014).

22. Mupepele, A. C., Walsh, J. C., Sutherland, W. J. & Dormann, C. F. An evidence assessment tool for ecosystem services and conservation studies. *Ecol. Appl.* **26**, 1295–1301 (2016).
