# Supplementary material for: A meta-analysis of the ecological and economic outcomes of mangrove restoration
Source: Nat Commun. 2021 Aug 19;12:5050. doi: 10.1038/s41467-021-25349-1 (PMC8376958; doi:10.1038/s41467-021-25349-1)
Supplement: Supplementary file 2 — Reporting Summary [file 41467_2021_25349_MOESM2_ESM.pdf]

nature research

Corresponding author(s): Jie Su

Last updated by author(s): Aug 3, 2021

Reporting Summary

Nature Research wishes to improve the reproducibility of the work that we publish. This form provides structure for consistency and transparency in reporting. For further information on Nature Research policies, see our Editorial Policies and the Editorial Policy Checklist.

Statistics

For all statistical analyses, confirm that the following items are present in the figure legend, table legend, main text, or Methods section.

n/a	Confirmed	
☐	☒	The exact sample size (*n*) for each experimental group/condition, given as a discrete number and unit of measurement
☐	☒	A statement on whether measurements were taken from distinct samples or whether the same sample was measured repeatedly
☐	☒	The statistical test(s) used AND whether they are one- or two-sided<br>*Only common tests should be described solely by name; describe more complex techniques in the Methods section.*
☐	☒	A description of all covariates tested
☐	☒	A description of any assumptions or corrections, such as tests of normality and adjustment for multiple comparisons
☐	☒	A full description of the statistical parameters including central tendency (e.g. means) or other basic estimates (e.g. regression coefficient) AND variation (e.g. standard deviation) or associated estimates of uncertainty (e.g. confidence intervals)
☐	☒	For null hypothesis testing, the test statistic (e.g. *F*, *t*, *r*) with confidence intervals, effect sizes, degrees of freedom and *P* value noted<br>*Give P values as exact values whenever suitable.*
☒	☐	For Bayesian analysis, information on the choice of priors and Markov chain Monte Carlo settings
☒	☐	For hierarchical and complex designs, identification of the appropriate level for tests and full reporting of outcomes
☐	☒	Estimates of effect sizes (e.g. Cohen's *d*, Pearson's *r*), indicating how they were calculated

Our web collection on statistics for biologists contains articles on many of the points above.

Software and code

Policy information about availability of computer code

Data collection	Quantitative data is extracted from the relevant peer-reviewed literature about the effect of mangrove restoration on different ecosystem functions, as well as the economic costs and benefits of mangrove restoration. The studies were identified through the ISI Web of Science Core Collection and Elsevier Scopus through two iterative literature searches. The first was on 22 December 2019 using generic words and second was on 30 June 2020 using a more refined keyword selection. Details are reported in the Methods (literature selection sub-section).
Data analysis	All meta-analyses and meta-regressions were performed using the package 'metafor' in R software (version 3.6.1). Details are reported in the Methods (Meta-analysis of restoration outcomes sub-section).

For manuscripts utilizing custom algorithms or software that are central to the research but not yet described in published literature, software must be made available to editors and reviewers. We strongly encourage code deposition in a community repository (e.g. GitHub). See the Nature Research guidelines for submitting code & software for further information.

Data

Policy information about availability of data

All manuscripts must include a data availability statement. This statement should provide the following information, where applicable:

- Accession codes, unique identifiers, or web links for publicly available datasets
- A list of figures that have associated raw data
- A description of any restrictions on data availability

The data that support the findings of this study are available in Figshare with the identifier https://doi.org/10.6084/m9.figshare.12901382.v6. The source data for plotting figures and tables can also be archived in the above link, except for Supplementary Table 5, Table 6, Table 8, and Supplementary Figure 12, which are directly created using R functions. The study quality assessment table is also available in the above link. The global distribution of mangrove can be obtained at UNEP-WCMC with the identifier https://doi.org/10.34892/1411-w728.

Field-specific reporting

Please select the one below that is the best fit for your research. If you are not sure, read the appropriate sections before making your selection.

☐ Life sciences ☐ Behavioural & social sciences ☒ Ecological, evolutionary & environmental sciences

For a reference copy of the document with all sections, see nature.com/documents/nr-reporting-summary-flat.pdf

Ecological, evolutionary & environmental sciences study design

All studies must disclose on these points even when the disclosure is negative.

Study description	In this study, we conduct a meta-analysis to quantify the outcomes of mangrove restoration and their magnitude, as a means of providing a quantitative estimate of mangrove restoration performance. We use a combination of statistical tools to examine (a) restoration outcomes for a range of biogeochemical, ecological and other functions across different comparative bases (i.e. restored mangroves vs. natural mangroves, naturally-regenerated mangroves, degraded mangroves, or unvegetated tidal flats), (b) the effect of diverse factors such as restoration age, approach, tree species, and region, on restoration outcomes, and (c) the economic costs and benefits of mangrove restoration.
Research sample	We followed the PRISMA protocol for study selection and inclusion in the systematic review and meta-analysis. The peer-reviewed literature was firstly searched through ISI Web of Science Core Collection and Elsevier Scopus using generic wording for restoration outcomes and impacts without restriction on publication year. The second search was conducted using a refined keywords of 30 ecosystem function categories and added possibilities that were identified after the first search. Details about the inclusion/exclusion criteria for publications are reported in Methods (literature selection sub-section). In total, 395 cases from 188 quantitative studies were identified, focusing on the effects of mangrove restoration on different ecosystem functions, as well as the economic costs and benefits of mangrove restoration. A case refers to one tree species with specific restoration age and same or similar environmental conditions, even when there are two or more measurements of restoration outcome. Overall, 962 observations from 88 studies covering 21 types of functions met the criteria to perform meta-analyses. For the meta-analysis each observations contained in the same peer-reviewed paper the performance of a restored mangrove for a given function(s), compared to one of four comparative bases (i.e. natural mangroves, naturally-regenerated mangroves, degraded mangroves, and unvegetated tidal flats) in the same area and environmental conditions. We also extracted 31 observations spanning ten types of economic benefits and 67 observations spanning six types of restoration costs to conduct the benefit-cost analysis. We used the five selection criteria for our study (see below Data exclusions). We formalized the quality appraisal of the individual studies following the criteria of Mupepele et al (2016) to assure the quality of studies included in the systematic review and meta-analysis.
Sampling strategy	We followed the PRISMA protocol for study selection and inclusion. The peer-reviewed literature was firstly searched through ISI Web of Science Core Collection and Elsevier Scopus using generic wording for restoration outcomes and impacts without restriction on publication year. The second search was conducted using a refined keywords of 30 ecosystem function categories and added possibilities that were identified after the first search. Details about the inclusion/exclusion criteria for publications are reported in Methods (literature selection sub-section). We formalized the quality appraisal of the individual studies following the criteria of Mupepele et al (2016) to assure the quality of studies included in the synthesis.
Data collection	J.S. conducted literature search and collected the data following the protocol designed jointly with A.G. Quantitative peer-reviewed studies about the effect of mangrove restoration on different functions, as well as the economic costs and benefits of mangrove restoration identified through ISI Web of Science Core Collection and Elsevier Scopus through two iterative literature searches. The first search was on 22 December 2019 using generic words and the second search was on 30 June 2020 using a refined keyword selection. Details are reported in the Methods (literature selection sub-section). We extracted mean, statistical variation (i.e. standard error, standard deviation) and sample size for restored and reference groups for each variable. When an original study reported the results graphically, we used webplotdigitizer (https://apps.automeris.io/wpd/) to extract data from figures. We also extracted the information of study site (i.e. country, coordinates of study area), restoration method, tree species, restoration age, and restoration species origin for each case. For the economic study, we extracted the quantitative values, specific type (e.g. economic benefit for climate regulation, plantation cost), and the valuation method.
Timing and spatial scale	The analyzed studies were published between 1992 and 2020, spanning a total of 22 countries and regions, mostly in East and Southeast Asia. Time and space distribution are reported in Supplementary Figure 4 and Figure 1 respectively.
Data exclusions	We established five selection criteria for our study. First of all, we excluded studies that focused on newly planted mangrove seedlings to ensure that the outcomes of mangrove restoration. In addition, we focused on empirical field studies and excluded experimental studies in laboratories, microcosms, tanks, greenhouses or pots to better compare with the reference groups (i.e. natural mangroves, unvegetated tidal flat, naturally regenerated mangroves, and degraded mangroves) in the same study site in the same study. Secondly, for the meta-analysis, we selected observations that contained in the same paper/study the performance of a restored mangrove for a given function(s), compared to one of four comparative bases in the same area and environmental conditions to ensure the proper assessment of restoration outcomes. In addition, we excluded study focusing on passive restoration as passive restoration without human intervention (i.e. natural succession) is regarded as a naturally-regenerated mangrove for comparison purposes. Furthermore, we did not consider studies assessing the ecological impacts of processes that interrupted mangrove restoration since our study focuses on the outcome of mangrove restoration. Finally, for the economic analysis, we only included studies specifically mentioning the monetary value of ecosystem services and the valuation method. All these exclusion criteria were a major component of the methodological design and were established from the beginning of the study from the authors.
Reproducibility	The methods of data collection and analysis are presented in the Methods section in detail and to enhance reproducibility the data

	supporting the results of this study are archived in figshare: 10.6084/m9.figshare.12901382.v6.
Randomization	Randomization is not applicable to a meta-analysis.
Blinding	Blinding is not applicable for this study because there is no experiments carried out.

Did the study involve field work? ☐ Yes ☒ No

Reporting for specific materials, systems and methods

We require information from authors about some types of materials, experimental systems and methods used in many studies. Here, indicate whether each material, system or method listed is relevant to your study. If you are not sure if a list item applies to your research, read the appropriate section before selecting a response.

Materials & experimental systems

n/a	Involved in the study	
☒	☐	Antibodies
☒	☐	Eukaryotic cell lines
☒	☐	Palaeontology and archaeology
☒	☐	Animals and other organisms
☒	☐	Human research participants
☒	☐	Clinical data
☒	☐	Dual use research of concern

Methods

n/a	Involved in the study	
☒	☐	ChIP-seq
☒	☐	Flow cytometry
☒	☐	MRI-based neuroimaging
